# The Sound and the Fury—Bees Hiss when Expecting Danger

**DOI:** 10.1371/journal.pone.0118708

**Published:** 2015-03-06

**Authors:** Henja-Niniane Wehmann, David Gustav, Nicholas H. Kirkerud, C. Giovanni Galizia

**Affiliations:** 1 Neurobiology, Universität Konstanz, Konstanz, Germany; 2 International Max-Planck Research School for Organismal Biology, Universität Konstanz, Konstanz, Germany; University of Arizona, UNITED STATES

## Abstract

Honey bees are important model systems for the investigation of learning and memory and for a better understanding of the neuronal basics of brain function. Honey bees also possess a rich repertoire of tones and sounds, from queen piping and quacking to worker hissing and buzzing. In this study, we tested whether the worker bees’ sounds can be used as a measure of learning. We therefore conditioned honey bees aversively to odours in a walking arena and recorded both their sound production and their movement. Bees were presented with two odours, one of which was paired with an electric shock. Initially, the bees did not produce any sound upon odour presentation, but responded to the electric shock with a strong hissing response. After learning, many bees hissed at the presentation of the learned odour, while fewer bees hissed upon presentation of another odour. We also found that hissing and movement away from the conditioned odour are independent behaviours that can co-occur but do not necessarily do so. Our data suggest that hissing can be used as a readout for learning after olfactory conditioning, but that there are large individual differences between bees concerning their hissing reaction. The basis for this variability and the possible ecological relevance of the bees’ hissing remain to be investigated.

## Introduction

Birds do it, bees do it, only educated fleas (presumably) don’t do it—they produce tones and sounds. These sounds are often used for intra- and interspecific communication, but besides that, animal sound production has also been used by researchers for the investigation of learning and memory in both animals and humans. Many different animals have been successfully conditioned to vocalise upon presentation of a stimulus: dogs learned to bark [1,2], rats to emit ultrasonic vocalisations in response to cues that predict foot shock [3,4], seals have been conditioned to emit clicks when facing visual cues [5,6], lemurs learned to call for food [7], also mynahs, shell parakeets [8,9] and chicks have successfully been trained [10], and even humans were conditioned to vocalise on certain cues (for an overview, see [11,12]).

Insects are missing in this list, and we chose the honey bee *Apis mellifera* to ask whether they can be conditioned to produce sounds upon presentation of an odour by classical conditioning with a mild electric shock as unconditioned stimulus. Honey bees are good model organisms for this question because they produce several sounds, such as buzzing, hissing and piping [13–15] and their sound production and detection might have biological significance (see [16] for an overview and discussion concerning the biological significance); furthermore, they are important model organisms to investigate invertebrate learning and memory.

**Fig 1:**
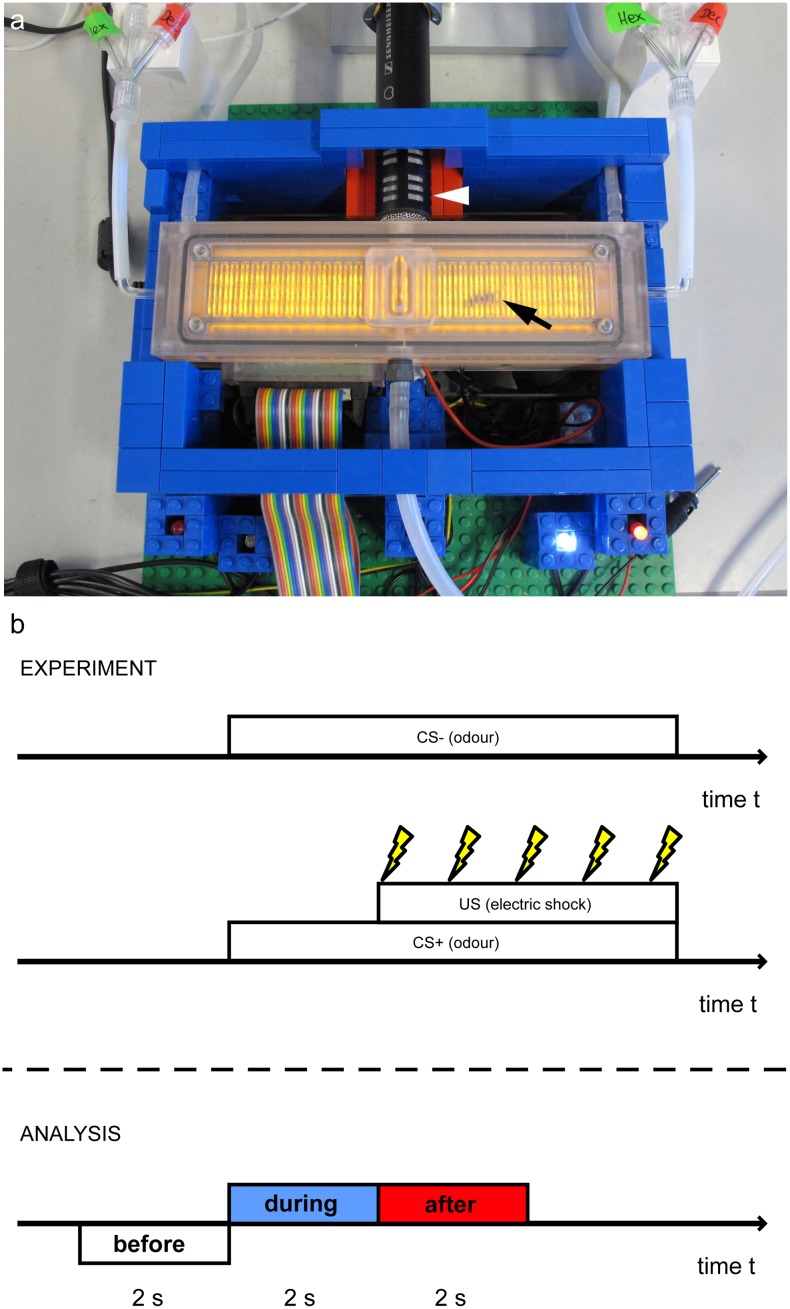
The Automatic Performance Index System and the protocol used. a) APIS is 148 mm long, 20 mm wide and 6 mm high, enabling unhindered walking on either floor or ceiling for the honey bee (black arrow). The interior is covered with an electrifiable metallic grid, and infrared-sensors constantly record the bee’s position. Odours were injected from either end of the chamber via computer-controlled valves. The bee’s hissing was recorded via the microphone attached to an opening in the chamber (top, white arrowhead). b) In order to quantify the bees’ hissing response, we analysed three sections of the recording for each trial: the two seconds before onset of the odours (before); the two seconds following the odour onset termed during (corresponding to the ISI in case of the CS+); and the first two seconds of the CS delivery after the ISI (after). Bees received 5 shocks (yellow lightening bolts) every 950 ms during the shock period in case of the CS+.

Honey bee learning and memory has been studied mostly in the laboratory using appetitive conditioning with the proboscis extension response (PER) paradigm [17,18] and also aversive conditioning with the sting extension response (SER, see [19]). We recently introduced APIS (Automatic Performance Index System, Fig. 1a) as a new tool to investigate learning in honey bees [20]. It consists of a walking arena, where the bee can freely move and where odours can be introduced and paired with electric shocks via an electric grid covering both floor and ceiling of the chamber. Bees quickly learn to avoid the side from which an odour is given after it has been associated with punishment (escape behaviour, see [20]). In those experiments, we observed that bees often produced a noise (which we termed “hissing", see discussion), presumably by moving their wings, during the electric shocks, and that many bees appeared to hiss also during the test upon presentation of the odour alone. These observations suggested that hissing is an innate response to noxious stimuli, and that it may be a conditioned response. It has been shown previously that bees start hissing (or as Collins and Rothenbuhler [21] termed it: begin a “flickering movement of the wings”) upon presentation of the alarm pheromone component isopentyl acetate and that this sound could be recorded using a microphone [22]. To our knowledge however, hissing has not yet been used as a readout for learning.

Our results demonstrate that hissing can be used as a conditioned response (CR) in honey bee conditioning, with approximately 40% of the bees showing this behaviour to the conditioned odour alone during a recall test five minutes later. We also find that hissing as CR and the bees’ being repelled by the odour as CR are independent behaviours: bees display either one, or both, upon smelling a conditioned odour. Our results offer new insights into honey bee behaviour and provide a new tool to investigate honey bee learning and memory.

## Material & Methods

### Honey bees

All experiments were conducted on *Apis mellifera* forager bees, which were caught at feeders placed nearby the hives on the roof of the University of Konstanz. A total of 104 bees were used in these studies.

### APIS—Automatic Performance Index System

APIS has been described in detail elsewhere [20]. In brief, it consists of a conditioning chamber (148 mm long, 20 mm wide and 6 mm high), covered with a metallic grid which can be electrified (Fig. 1a). Bees can walk unhindered on either floor or ceiling. Infrared (IR) sensors detect and record the position of the bee continuously and allow monitoring the bees’ behaviour as well as side-specific delivery of both odours and shocks. APIS allows controlling the timing of odours given, and the timing of electric shocks delivered to the animal. In this study, we added sound recording to APIS (see below).

Bees were differentially conditioned to two odours in a classical conditioning paradigm.

#### Odours

150 μl of diluted odour (1-hexanol and 1-decanol diluted 10^-3^ in mineral oil, all chemicals were obtained from Sigma Aldrich, Taufkirchen, Germany, >98% purity) were applied to absorbent cellulose rectangles (Sugistrips, Kettenbach GmbH & Co. KG, Eschenburg, Germany) placed at the distal end of 2 ml plastic syringes (Henke-Sass, Wolf GmbH, Tuttlingen, Germany). Odours were delivered to the chamber from either side using computer-controlled valves. Continuous air suction at the centre and the ends of the conditioning chamber removed the odours from APIS.

#### Differential classical odour conditioning

Odour stimulus duration was 6 seconds. For the conditioned stimulus (CS+), a mild electric shock (unconditioned stimulus, US) was administered 2 s after odour onset for a total duration of 4 s. The US consisted of five 200 ms shocks (10 V) given every 950 ms (Fig. 1b). A non-punished odour (CS-) was given for 6 seconds without any electrical stimulation.

All odours were delivered at the side where the bee was located. The US was delivered on both sides of the chamber.

The inter-trial interval (ITI) was 36 s. Bees were exposed to 8 training trials (4 CS+, 4 CS-) in pseudorandomised order (ABBABAAB or BAABABBA). After conditioning, the lights in the chamber were shut off, but the bee remained in the chamber. After five minutes the lights were switched on again, and after additional 30 s bees were tested for their short-term memory in a 4-trial test, in which both odours were presented with an ITI of 36 s without shocks in a pseudorandomised order (ABBA or BAAB). The bee’s position was continuously sampled by IR sensors and written to a log-file.

After the experiment, the bee was removed from the chamber, chilled in a freezer and sacrificed in 70% ethanol. The interior of the chamber was cleaned with ethanol to remove possible contaminations. The chamber was rinsed with hot water and detergent every day.

### Audio recordings and analysis

Honey bee hissing was recorded using a Sennheiser ME 64 microphone (cardioid pick-up pattern, frequency response 40–20.000 Hz ± 2.5 dB) attached to a computer sound card. All recordings were made as uncompressed 16-bit PCM audio at a sampling rate of 44.1 kHz using the software Audacity [23], and stored in WAV format. The microphone was placed at an outlet at the chamber’s centre (see Fig. 1a, white arrow). Thus, the microphone was between 1.5 and 9 cm away from the bee (depending on the bee’s position in the chamber). This experimental setup allowed recording of hissing occurring anywhere in the chamber.

### Data and statistical analysis

We used a customised script written in R [24] to extract and analyse the movement traces of the bees during the experimental stimulation period and to identify the bees’ hissing in the sound recordings.

Sound data was read into R using the package “seewave” [25] and the “ReadWave” function of the package “tuneR” [26]. The sound data was then synchronised with the position of the bee and information on the stimuli from APIS (S1 Fig.).

Hissing was determined by creating a spectrogram (function “spectro” of the “seewave” package; Hanning-window, window length of 2048 points, corresponding to 46 ms) and evaluating the summed intensities (in dB) at different frequency ranges (see S2 Fig. for an example spectrogram).

Ambient noise and the sound from the in- and outflowing air into and from the conditioning chamber were mostly confined to frequencies below 15 kHz, while hissing reached up to about 20 kHz. Therefore, we identified hissing by the summed intensities for frequencies higher than 15 kHz. Clicking of valves and some other ambient sound events during recording had a frequency range close to hissing, we excluded those based on high summed intensities in the spectrogram in the frequencies above 20.05 kHz and their length: only events with a minimum of 3 consecutive spectrogram time bins classified as hissing (roughly equal to more than 0.09 s) were considered as actual hissing (see S2 Fig. and S3 Fig. for a histogram). Hissing of all bees was quantified and plotted (S4 Fig.).

We analysed three sections of the recording for each trial: the two seconds before onset of the odours (before); the two seconds following the odour onset (during, this time interval corresponds to the interstimulus-interval (ISI) in case of the CS+); and two seconds following the ISI-phase of the odour stimulation (after, see Fig. 1b for a graphical depiction). During the after section, bees received shocks in case of the CS+.

Occurrence of hissing was quantified as a binary value. If a bee did not hiss at all during one such span, it received a 0, if it did hiss, it received a 1. We calculated learning curves as the fraction of hissing bees. From the responses to the CS+ and the CS-, a learning index (LI) was calculated for each bee and each test trial by subtracting the bee’s response to the CS- from the response to the CS+. The mean of the 104 bees’ individual LIs was calculated to obtain a LI for each section (before, during, after).

Example movement traces can be found in S1 Fig., a-c. To quantify the bees’ responses towards the injected odours, we assessed the performance variable “Escape” [20] for each odour stimulus. Whenever a bee was repelled by the odour and located in the chamber’s half opposite to the odour stimulus 2 seconds after the stimulus onset, it was counted as a successful “Escape”. We chose the variable “Escape” as a robust behavioural response. It represents a binary description of the bees’ behaviour and thus allows a direct comparison with the binary hissing responses. The outcome is comparable to the well-known analysis of PER data or SER data [20].

For statistical testing of learning scores, we used analyses of variance (ANOVAs) and appropriate post-hoc comparisons on the data. It is permissible to use ANOVA on dichotomous data when there are at least 40 degrees of freedom [27,28]. These conditions were met by our experiments. All analyses were performed using R [24] or Statistica 6.1 (StatSoft Europe GmbH, Hamburg, Germany). 95% Clopper-Pearson confidence intervals [29] were calculated for the different stimuli groups. The analysis based on ANOVA corresponds to common practice in learning studies and allows for direct comparison with other papers. However, from a statistical point of view, modelling binary data is a more appropriate approach for binary behavioural observations [30] Therefore, we additionally used a generalised linear mixed model (glmm) with a binomial distribution setting (logistic regression). We fitted the response data (hissing and escapes) using the “glmer” function of the “lme4” package [31] in R. Hissing and escape response served as binary response variables, while trial (1–4), conditioned stimulus (CS+ or CS-) and odour (hexanol or decanol) with interactions were included as fixed variables. The bee identity served as a random varaible to account for the repeated measurements. Fitted means and 95% credible intervals of the quantified variables were estimated using the “fixef” function and the “sim” function from the R-packages “lme4” [32] and “arm” [33], respectively. Direct hypothesis tests were executed by calculating the probability of the fitted mean for the CS- response occurring within the posterior distribution of the CS+ response for a given trial. S5 Fig. shows the fitted means for hissing, escape and the combination of these variables, S6 Fig. shows a comparison between fitted values and the observed means. Diagnostic plots for the goodness of fit can be found in S7 Fig. There were no qualitative differences between the statistical tests based on ANOVA and on binary data models.

## Results

Bees underwent an 8-trial differential conditioning paradigm consisting of four reinforced trials (one odour paired with electric shocks, CS+) and four non-reinforced trials (another odour not paired with shocks, CS-, see Fig. 1b upper part) arranged in a pseudorandom order. A total of 104 bees were conditioned and both their hissing as well as their locomotor response to the odour were recorded. The bees were equally distributed over the two odours used: 52 bees were conditioned with hexanol as CS+, 52 with decanol. When entering the chamber, bees usually started to explore the conditioning chamber by walking it end to end.

### Hissing response

Most bees responded to the electric shock with hissing: 101 of the 104 bees tested hissed at least once during the conditioning phase (97.1%). The three bees that never responded during conditioning also did not respond during testing.

**Fig 2:**
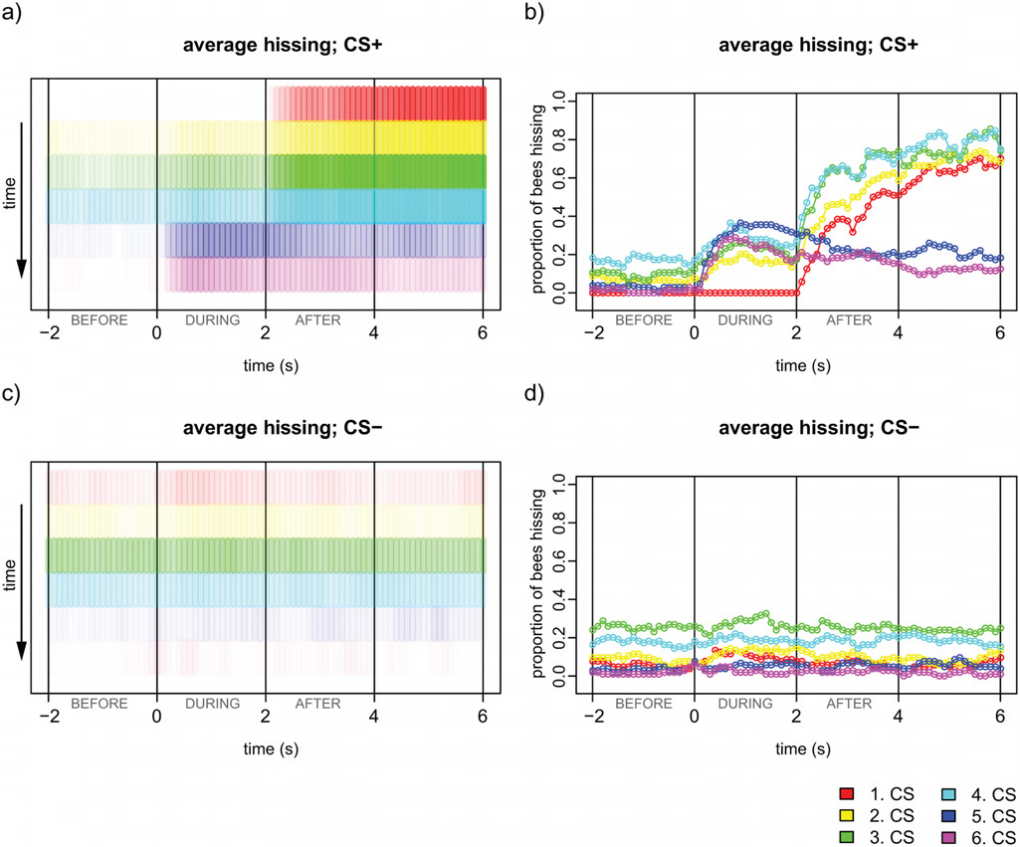
Average hissing for CS+ and CS- with respect to timing. a, c: Colours indicate the trial (see legend, 1.-4. CS+ are paired with electric shock at 2s (after)) and their intensity the proportion of bees hissing for the CS+ (a) and the CS- (c). b, d: Proportion of bees hissing during the three different sections analysed for both the CS+ (b) and the CS- (d). Same data as in a, c. 0 seconds represents CS onset. The plots show that during CS-, less bees hiss than during CS+. Hissing in the during section increases over trials and bees tend to stop hissing in the CS+ recall test (5. CS and 6. CS), when no shocks are given. n = 104 bees. Binning 100 ms.

**Fig 3:**
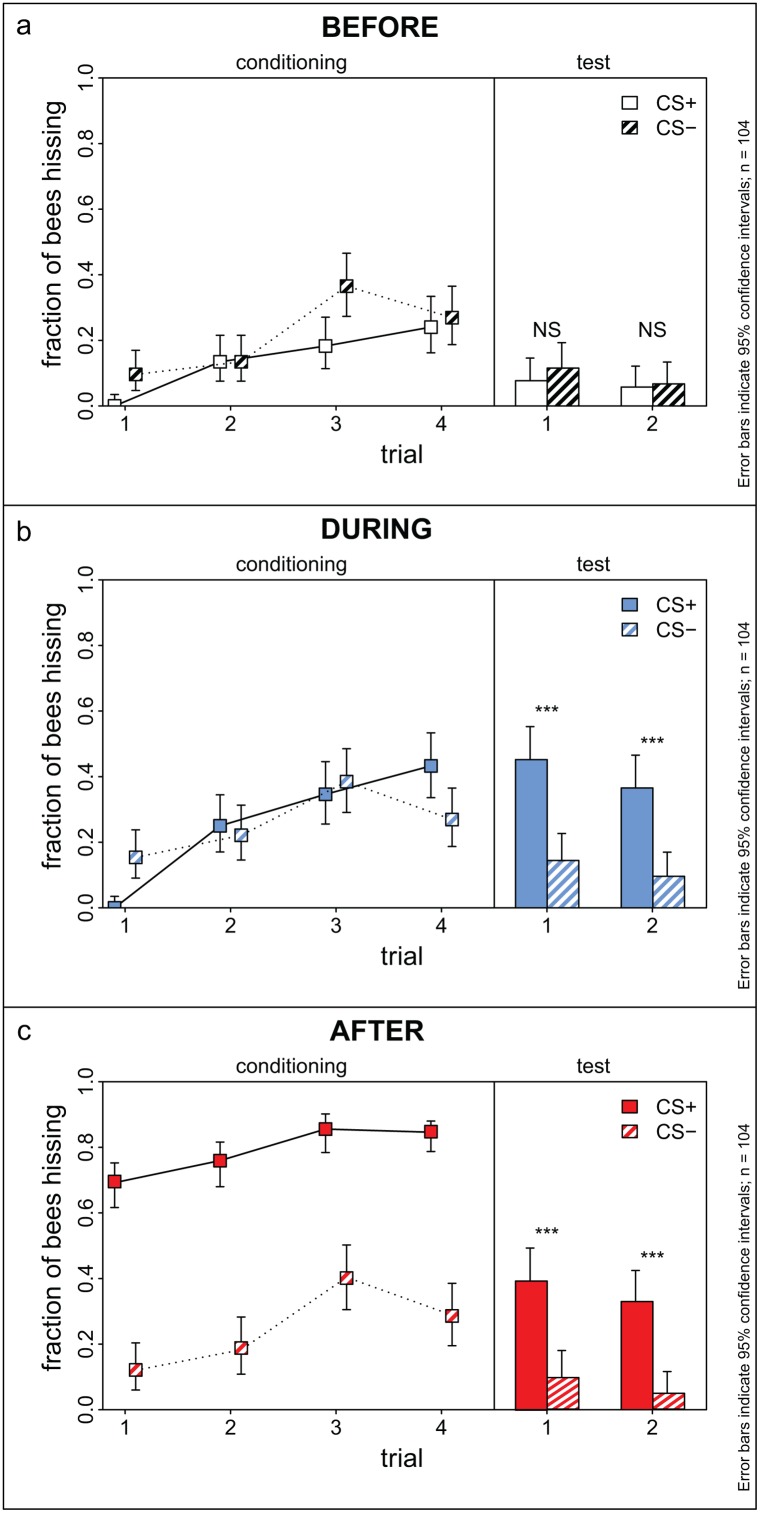
Hissing of honey bees during differential conditioning and recall test. Left: Acquisition curve. Right: Recall test five minutes after the end of conditioning. Shown are the three sections before and during odour presentation: a) before section, b) during section, c) after section. Solid lines and bars: CS+, dashed lines and shaded bars: CS-. Bees do not respond differently to CS+ and CS- before onset of the odour (a), but after onset of the odour, they respond more often to the CS+ than to the CS- with hissing (b, c). There is no clear distinction between CS+ and CS- during the conditioning phase in the during section (b, left panel, only the first and the last conditioning trial show a significant difference), bees hiss more often during the shocked phase (after section) of the CS+ (c, upper curve) compared to the CS- (c, lower curve). Asterisks indicate statistical difference, RM-ANOVA followed by Fisher LSD-post hoc test, p<0.0001. Error bars show the 95% confidence interval.

Learning acquisition of hissing was evaluated using three temporal sections: the two seconds before odour onset (before), the two seconds during ISI where only the odour was present (during) and the first two seconds after the ISI, when shocks were administered in case of the CS+ (after, see also Fig. 1b, lower part). As Fig. 2 and Fig. 3 show, bees typically responded neither before odour onset nor during the ISI for the first trial (CS+ before 0%: CS- before 9.6%, CS+ during 0%, CS- during 16.3%), whereas bees started hissing at shock onset (CS+ after: 69.2%, CS- after 12.5%, red bars/curve in Fig. 2a and b). The bees that responded to the CS- in the before and during section in the first trial (in total 18 animals, see Fig. 2c and Fig. 3a, b, dashed lines and shaded squares) all underwent an ABBA-conditioning regime (with A denoting CS+). These bees either were aroused to hiss for long periods (those 10 bees active in the before section), or generalised from the CS+ to the CS- by responding to the presentation of the new odour with hissing (during). Generalisation was symmetric for odour: 9 of these 18 animals had hexanol as CS+, 9 decanol.

There was no difference between CS+ and CS- in the before section of the last conditioning trial: 25.0% of the bees hissed before the onset of the last reinforced CS+ and 27.8% hissed before the onset of the corresponding CS- (Fig. 3a, RM-ANOVA, F_1, 102_ = 0.52, P = 0.47).

The during response for the last conditioning trial was 42.3% to the CS+ and 27.8% to the CS- (Fig. 3b). This difference was statistically significant (RM-ANOVA; F_1, 102_ = 8.97, P = 0.003), indicating that bees hissed more to the CS+ than to the CS-;. In the after section of the last conditioning trial, 84.6% of the bees hissed during the CS+ while being shocked, while only 28.8% of the bees hissed during the same section of the CS- (Fig. 3c, RM-ANOVA; F_1, 102_ = 120.82, P<0.0001).

5 minutes after the end of conditioning bees were tested two times for each odour without reinforcement. 53.8% (56 out of 104 bees) responded to at least one CS+ with a hiss (during and after section combined since no shocks were given in the after section).

In the during sections of the test, 45.2% of the bees hissed upon presentation of the first CS+ and 36.5% to the second CS+, whereas only 15.4% and 10.6% responded to the first and the second CS-, respectively. These response magnitudes were comparable to the last training trial (Fig. 3b, right part). The difference between CS+ and CS- was statistically significant (RM-ANOVA, odours x stimulus sequence, F_3, 288_ = 23.59, P<0.0001), indicating that the bees clearly had learned the two odours, and that this was displayed in their hissing response. The response to the odours was similar for the during and the after section (comparing CS+ and CS-, respectively, using an RM-ANOVA followed by Fisher LSD post-hoc test, P>0.05, Fig. 3b, c). This suggested that bees did not learn the timing of the shock onset.

To assess the consistency of the bees behaviour, we analysed the individual bees’ responses to the first and second CS+ for the during section in the test. 33 bees (31.7%) responded to both stimuli (consistent learners), 48 bees did not respond (consistent non-learners), 14 bees hissed only for the first CS+ and not to the second (memory extinction), and 5 bees responded only to the second CS+ ("inconsistent" bees).

Eight bees (7.7%) investigated hissed in the during sections of all CS+ (except the first CS+, i.e. “single-trial learners”), whereas only 2 bees (1.9%) responded to all CS- (also excluding the first CS-). Interestingly, the bees that hissed for all CS- were not the same bees that hissed for all CS+. 24 bees (23.1%) hissed to at least 4 of the 5 CS+—when taking into consideration only the 52 bees that responded during the during section of at least one of the CS+ test trials, almost half of these bees (46.1%) were consistent with respect to their hissing behaviour.

#### Hissing Learning Index

**Fig 4:**
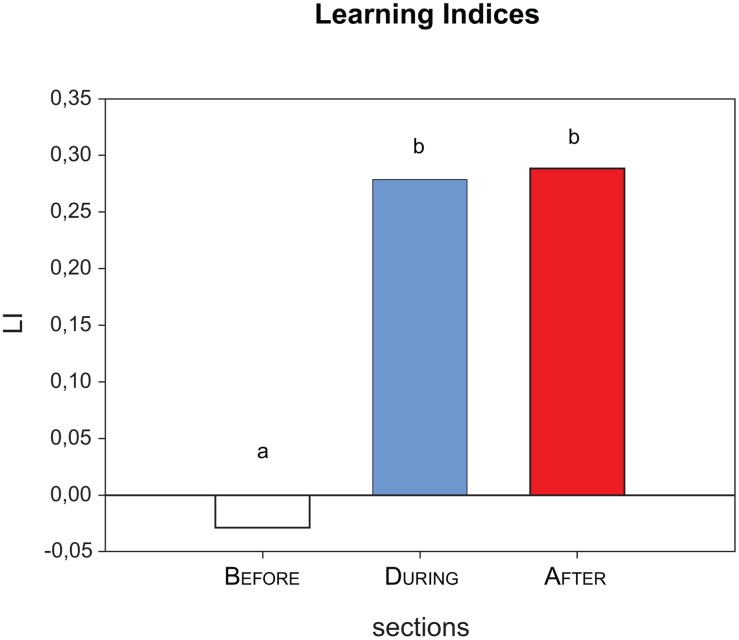
Learning indices. The learning indices (LI) were averaged across the two test trials for data shown in Fig. 3a-c. The LIs clearly show that in the before section, the CS+ and the CS- trials did not differ, whereas for both during section and after section, bees responded more to the CS+ than to the CS- (positive LIs). There was no statistically significant difference between during section and after section, indicating that bees did not alter their hissing behaviour by much at the predicted timing of the shock. Letters indicate statistical difference, RM-ANOVA followed by Fisher LSD-post hoc test, p<0.0001. n = 104 bees.

A higher learning index LI corresponds to a stronger hissing towards the CS+ compared to the CS-, thus higher discrimination ability of the honey bees. The LI averaged across the two test trials (Fig. 4) varied between the three sections: there was no difference between CS+ and CS- trials previous to the onset of odours (reflected by the LI being close to zero for the before section, -0.02). In the period after odour onset, more bees hissed to the CS+ compared to the CS-, resulting in LIs of 0.27 (during) and 0.28 (after, Fig. 4). The LI was different for the section before odour onset as compared to the sections after odour onset (during and after), but there was no statistically significant difference between the two latter sections concerning the LIs (RM-ANOVA followed by a Fisher post-hoc comparison, F_2, 412_ = 48.19 with P<0.0001 for the comparison before vs. during/after and P>0.05 for the comparison during vs. after).

#### Effect of odours and sequence

Both odours were balanced as CS+, and there was no significant difference between the two odours with respect to hissing during the test phase in the during section or in the after section (RM-ANOVA_during_: F_1, 96_ = 0.87 with P = 0.352, RM-ANOVA_After_: F_1, 96_ = 2.02 with P = 0.158, see also S8 Fig. for the learning curves). There was also no statistically significant difference with respect to the two sequences used (RM-ANOVA_during_; F_3, 96_ = 0.31, P = 0.815, RM-ANOVA_After_; F_3, 96_ = 0.53 with P = 0.663).

### Escape response

We defined the “Escape” response as moving away from the odour within the first two seconds after odour onset, crossing the midline and not returning to the side the odour was injected on. We excluded five bees from the analysis of their locomotion behaviour because they stopped moving, suggesting that they were exhausted. Thus, 99 bees were analysed.

**Fig 5:**
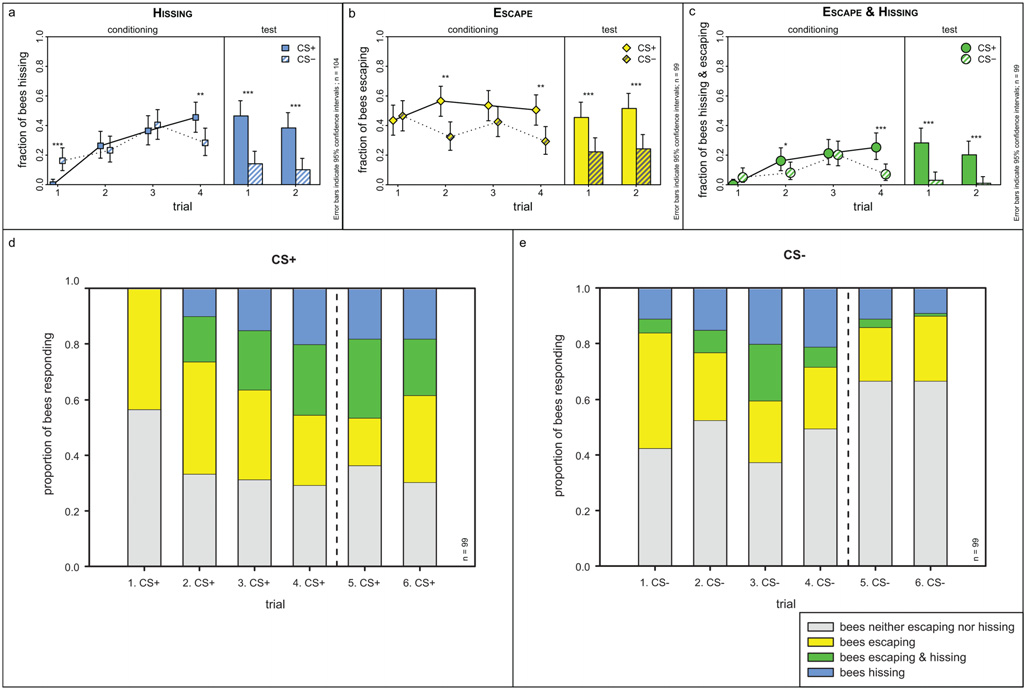
Behavioural data of the differentially conditioned honey bees: hissing, escapes from odour and combination of these two. a) Hissing of bees during odour presentation (during section)—same data as in Fig. 3b. b) Escapes by the bees from odour. Left panel: acquisition, right panel: recall test. Bees learned to escape from the odour during conditioning and escaped with a higher probability from the CS+ than from the CS- from the second trial on. This difference remained stable throughout the recall test. c) Proportion of bees that both hissed and escaped from the odour. Although only few bees exhibited both behaviours, there is a very clear difference between CS+ and CS-: almost no bees responded with hissing and escape to the CS- (2%), whereas 24.2% of the bees responded to the CS+ with both behaviours at the same time during the test. Solid lines and bars: CS+, dashed lines and shaded bars: CS-. Asterisks indicate statistically significant difference (RM-ANOVA followed by Fisher LSD-post hoc test, p<0.0001); error bars denote 95% confidence interval. d, e) Proportion of bees hissing, escaping, doing both or none of these responses upon presentation of CS+ (d) and CS- (e). The dashed line separates the conditioning phase (trial 1–4) from the recall test phase (trial 5 and 6).

During the first conditioning trial, bees escaped to the same degree from the CS+ (43.4%) and from the CS- (46.4%, RM-ANOVA; F_1, 97_ = 0.25, P = 0.618), but already at the second conditioning trial, more bees escaped from the CS+ compared to the CS- (RM-ANOVA; F_1, 97_ = 15.03, P<0.001). The effect waned in the third conditioning trial, but the last conditioning trial was again statistically significant (RM-ANOVA; F_1, 97_ = 8.32, P = 0.0048, Fig. 5b, left panel).

Also during the recall test five minutes after the end of conditioning significantly more bees escaped from the CS+ than from the CS-. This difference was similar for both recall test trials (Fig. 5b, right panel, RM-ANOVA, F_3, 291_ = 10.81 with P<0.0001): while 45.5% and 51.5% of the bees escaped from the CS+ (first and second trial, respectively), only 22.2% and 24.2% of the bees escaped from the CS- (first and second trial, respectively).

As with hissing, we could not find any significant difference between the two odours with respect to escape probability (RM-ANOVA; F_1, 97_ = 0.17 with P = 0.677, see also S9 Fig. for the respective learning curves). There was also no statistically significant difference in escapes with respect to the sequences used for conditioning (RM-ANOVA; F_1, 97_ = 0.76, P = 0.386).

### Combination of hissing and escape

Are hissing and escape behaviour linked, or are they independent? Response with either escape *or* hissing during the test trials showed significantly more bees responding to the CS+ compared to the CS- (1. test trial: 63.6% vs. 33.3%, RM-ANOVA; F_1, 97_ = 22.098, p<0.001, 2. test trial: 69.7% vs. 33.3%, RM-ANOVA; F_1, 97_ = 36.295, p<0.001, S10 Fig.). These figures are higher than either hissing or escape alone, suggesting an additive effect. Indeed, not all bees which escaped also hissed and vice versa (for both CS+ and CS-, Fig. 5). During the recall test, approximately 20% of the bees hissed without escaping (S11 Fig., with data on all trials). In the last conditioning trial, almost as many bees hissed in the during section when confronted with the CS+ without escaping as bees which did both, hissing and an escape (19.2% vs. 24.2%, Fig. 5c and d). Interestingly, for the after section of the first CS+, 40.4% of the bees hissed upon presentation of the electric shock without escaping. This might indicate that hissing is a rather spontaneous response to noxious stimuli, whereas escaping involves more complex tasks such as orientation and locomotion into the right direction which might take longer to establish. Together, these results suggest that movement and hissing are independent motor programs that can be combined but need not be.

## Discussion

Honey bees can be conditioned to respond to an odour with a hissing response by pairing the odour with an electric shock. To our knowledge, this is the first time an insect has been conditioned to produce sound, although similar experiments have been undertaken in other animals, using both operant conditioning [e.g. in dogs and lemurs, 1,2,7] and classical conditioning (for example in rats which produce ultrasonic vocalisations when faced with a stimulus predicting an electric shock [3], which is rather similar to the experimental design described here).

### Hissing or buzzing?

Honey bees produce a variety of sounds using their wings, e.g. for pollen collection, defence, recruiting foragers during the waggle dance and during swarming. Both “hissing” and “buzzing” have been used for somewhat similar sounds produced by bees. “Buzzing” has been used mainly to describe the bees’ sound production during the waggle dance [34–36], foraging [37] or during swarming [38–44]. Buzzing is used as an attraction signal also by other bee species [45], and likely is an evolutionary old signal. In some publications, “buzzing” has also been used to describe defensive behaviour in honey bees [22,46,47], but generally, “hissing”, or “shimmering” is used in this context [48–50]. Therefore we use “hissing” to describe the bees’ response to noxious stimuli. “Hissing” has also been used in other species, e.g. for aggressive behaviour in, rats [51], rattlesnakes [52] and pine snakes [53], chickadees [54], lizards [55], cats [56,57] and hissing cockroaches [58]. While the proposed distinction between the two terms is purely based on behavioural context, further analyses may reveal physical differences between honey bee “hissing” and “buzzing” (e.g. in terms of frequency, sound pressure level, or duration). To our best knowledge, bees use their wings to create the hissing sound analysed in this paper.

### Hissing as a readout for learning

We used hissing as a behavioural readout for aversive conditioning in honey bees. We found a learning rate of 40.8% (1^st^ and 2^nd^ test trials combined), which is slightly lower than the escape rate with 48.4%. Compared to other paradigms, the learning rate is relatively low: appetitive PER-conditioning results in learning rates of 80% or higher (see [17,18] for examples), and aversive SER-conditioning of about 60% [19]. Although the learning rate is rather low, bees do not respond by chance with a hiss: comparing the before section with the during section shows that most bees hiss only as a reaction to the odour/shock and only hiss occasionally when not prompted.

Just like PER and SER, hissing is an innate response of the bee upon presentation of a stimulus, whereas escape (also because of its strong motor component) might be more comparable to the acquisition of flower handling skills in generalist pollinators as shown in bumble bees [59]. While the hissing learning rate in this first study is low compared to PER and SER, it might be improved by changing experimental parameters: switching the massed to a spaced conditioning, or increasing the number of trials. Generally, the trial numbers and conditioning procedure strongly influence the learning rate [60]. Although massed conditioning has been shown to form a stable odour memory (at least in PER-conditioning [61]), the bees’ learning in APIS might be incomplete: the memory might be constantly “overwritten” during conditioning. Bees might need a break for consolidation to show the discrimination between CS+ and CS- which we saw during the test phase. Additionally, the electric shocks might induce stress and a constant state of alertness, preventing them from showing discriminatory behaviour. In this case their response threshold may be lowered, resulting in an overall elevated tendency to hiss. This view is supported by our data: bees conditioned starting with the CS- hissed less between the begin of conditioning and the first CS+ than during similar time spans in the five minute break between conditioning and recall, indicating a higher “spontaneous” hiss rate after training (see S4 Fig., lower part).

### Hissing and locomotion

When combining the two readouts “hissing” and “escape”, one quarter of bees responded upon presentation of the CS+ (24.2%). This very low learning rate is compensated by the low level of generalisation ("false-positives", i.e. responding to the CS- with both an escape and hissing): only 2.0% of the bees responded in that way, even though 23.2% of bees responded with an escape, and 12.9% with hissing. This indicates that bees which hiss and escape upon presentation of an odour can distinguish the odours precisely and are not likely to generalise.

Our results show that not every bee that hisses also escapes, and vice versa. For example, a significant number of bees hissed but did not escape (Fig. 5d, e)—it remains to be investigated why and how these two behaviours interact.

Hissing has been shown to act as a warning cue aimed at an attacker [62], and it has been shown that handling and stress affects the bees’ levels of hemolymph dopamine, octopamine, and serotonin, and influence their hissing responses [22,63]. Further investigations are needed to address the dependencies of escape, hissing and the bees’ internal state, to evaluate the social value of the two behaviours shown here and to investigate the possible relationship of sociality and sound production of bees in more detail.

### Outlook

We were able to show that hissing can be used as readout for learning in honey bees, adding an insect to the list of animals which can be conditioned to produce sound. So far, we only analysed the bees’ responses in a binary manner (hissing/no hissing). Future experiments need to look into temporal differences between the hissing (e.g. in duration between CS+ and CS-, see S12 Fig. for an estimation) or intensity changes over the course of the experiment. The adaptive significance of hissing vs. escape also needs dedicated experiments. For example, it would be intriguing if hissing could be used as a US itself (meta-conditioning), or as a modulator of behaviour (if hissing was indeed used for communicating danger, hearing another bee hiss may induce behaviours such as sting extension). It has been shown that exposure to alarm pheromone impairs appetitive learning in honey bees [64], and hissing could act as a “far-distance” signal in the hive to alert other bees. Armbruster at the beginning of the last century already speculated that honey bees communicate with sounds and that honey bees’ hissing or buzzing might affect other bees, but was very sceptical about this [14]. Further investigations are needed to assess the biological relevance of hissing in a natural context. The sounds produced by bees have many secrets left to reveal.

## Conclusions

Our work shows that the hissing of honey bees after conditioning can be used as readout for the animals’ learning and memory. As hissing is independent from other behavioural responses (e.g. escape), our results suggest that hissing is behaviourally similar to reflex-like behaviours such as the extension of the bees’ proboscis or it’s sting. We propose that honey bee hissing might serve as an intraspecific signal to warn conspecifics.

## Supporting Information

S1 Fig Movement traces in APIS. This figure shows the movement trace within APIS of three example bees during conditioning (upper part) and test (lower part). Times of odour delivery (on the side the respective bee was located on) and electric shock (given on both sides of the chamber) are indicated, grey bars indicate hissing (see legend). a) The bee shown in this example hissed very scarcely during conditioning and generalised during the test towards the first CS-. We observed very little generalisation in our data, so hissing seems to be a very robust readout (compare also Fig. 3 and S5 Fig. and S6 Fig.). b) Example trace of a bee hissing spontaneously (i.e. without preceding odour stimulus) during the test. This behaviour was not seen very often, but occurred occasionally. c) The bee shown here hissed strongly during conditioning and perfectly during the test. Note that during the test the bee stops hissing after the first seconds of odour presentation, anticipating the shock and stopping the behaviour after its expectations were not fulfilled.
(PDF)

S2 Fig Example spectrogram. This example spectrogram belongs to the bee shown in S1c Fig. These spectrograms were used to identify hissing (see Material & Methods). The clicks of the opening and closing valves can be clearly seen as very sharp lines spanning from 0k to 18k (solid red arrow), whereas the hissing was more blurry, longer and starting only at around 4k (see trace underneath). Valves of neighbouring setups could be also recorded (dashed red arrow). Blue arrowhead marks end of conditioning, yellow arrowhead begin of recall test.
(PDF)

S3 Fig Histograms of honey bee hissing. a) Histogram of the durations of all sounds recorded and initially classified as hissing before removing hisses shorter than ca. 0.09 s, which were regarded as valve clicking sounds and other events not related to bees’ hissing. b) Histogram of hissing lengths. Bin size for both plots 0.05 s. n = 104 bees.
(PDF)

S4 Fig Hissing of all bees conditioned and tested during the course of the experiment. Grey double lines indicate odour presentations. In the upper part are all bees that underwent an ABBA conditioning paradigm (CS+ first), in the lower part all bees that underwent a BAAB conditioning paradigm (CS- first). Before the first CS+, few bees hiss, whereas after conditioning (300 to 600 ms), the bees’ general tendency to hiss is increased. Very few bees hissed constantly, and very few bees never hissed during the experiment (“Zombees”, one example ─ bee #26 ─ can be found at the transition from yellow to green). n = 104 bees.
(PDF)

S5 Fig Modelled data from the analysis of escapes and hissing using the generalised linear mixed model. Learning studies often use ANOVA to analyse the data, however, modelling binary behavioural data is a more appropriate approach. Hissing and escape response served as binary response variables, while trial, conditioned stimulus (CS+ or CS-) and odour (hexanol or decanol) with interactions were included as fixed effects. Bee identity served as random effect to account for the repeated measurements. The credible interval for the first data point (CS+ first trial) in a) and c) spans from 0 to 1 because no observations could be made for this event. The model cannot estimate data and interval without any incident to start the estimation from. Asterisks denote statistical difference (p<0.001).
(PDF)

S6 Fig Fitted values vs. observed means. To compare the observed means (“obs., denoted by crosses) and the modelled values (“fit”), both were plotted in one figure, showing that the model generally underestimates the bees’ hissing response (see difference in a), whereas it matches the escape response (see b). Combining the two variables leads to a slight underestimation of the model compared to the observed data (c).
(PDF)

S7 Fig Goodness of fit plots for hissing and escape data fitted by binary logistic regression. Observed vs. fitted values (open circles) are plotted with class-wise means and 95% confidence interval for the observations (orange dots and bars). The dotted line indicates perfect coincidence between the observed data and the model prediction. The confidence intervals of the class-wise means mostly span the diagonal line, which indicates that the logistic regression model predicts the data tolerably well.
(PDF)

S8 Fig Results of the differential conditioning of honey bees and recall test for the two odour configurations used. a-c) Decanol was used as CS+ and hexanol as CS-. d-f) Hexanol was used as CS+ and decanol as CS-. There was no statistically significant difference between the two odour configurations detectable.
(PDF)

S9 Fig Honey bee escapes with respect to odours. No statistically significant differences could be found when analysing the bees’ escape behaviour with respect to the odour configuration. Bees which were conditioned to decanol as CS+ (a) as well as bees that were conditioned to hexanol as CS+ (b) distinguished between CS+ and CS- after conditioning to the same extent.
(PDF)

S10 Fig Proportion of bees responding with hissing or escaping. During the test, responses to the CS+ are higher as compared to the CS-. Error bars denote 95% confidence interval.
(PDF)

S11 Fig Honey bees hiss without escaping, also while being shocked. Error bars denote 95% confidence interval.
(PDF)

S12 Fig Hissing duration and hissing onset relative to odour, as estimated by our hissing-recognising R-code. Most hisses are less than 1 s long, only very few hisses are longer than 10 seconds. Duration increases (not very surprisingly) with shock onset. n = 104 bees.
(PDF)
