# Supplementary material for: The Sound and the Fury—Bees Hiss when Expecting Danger
Source: PLoS One. 2015 Mar 6;10(3):e0118708. doi: 10.1371/journal.pone.0118708 (PMC4351880; doi:10.1371/journal.pone.0118708)
Supplement: S1 Fig — This figure shows the movement trace within APIS of three example bees during conditioning (upper part) and test (lower part). Times of odour delivery (on the side the respective bee was located on) and electric shock (given on both sides of the chamber) are indicated, grey bars indicate hissing (see legend). a) The bee shown in this example hissed very scarcely during conditioning and generalised during the test towards the first CS-. We observed very little generalisation in our data, so hissing seems to be a very robust readout (compare also Fig. 3 and S5 Fig. and S6 Fig.). b) Example trace of a bee hissing spontaneously (i.e. without preceding odour stimulus) during the test. This behaviour was not seen very often, but occurred occasionally. c) The bee shown here hissed strongly during conditioning and perfectly during the test. Note that during the test the bee stops hissing after the first seconds of odour presentation, anticipating the shock and stopping the behaviour after its expectations were not fulfilled. (PDF) [file pone.0118708.s001.pdf]

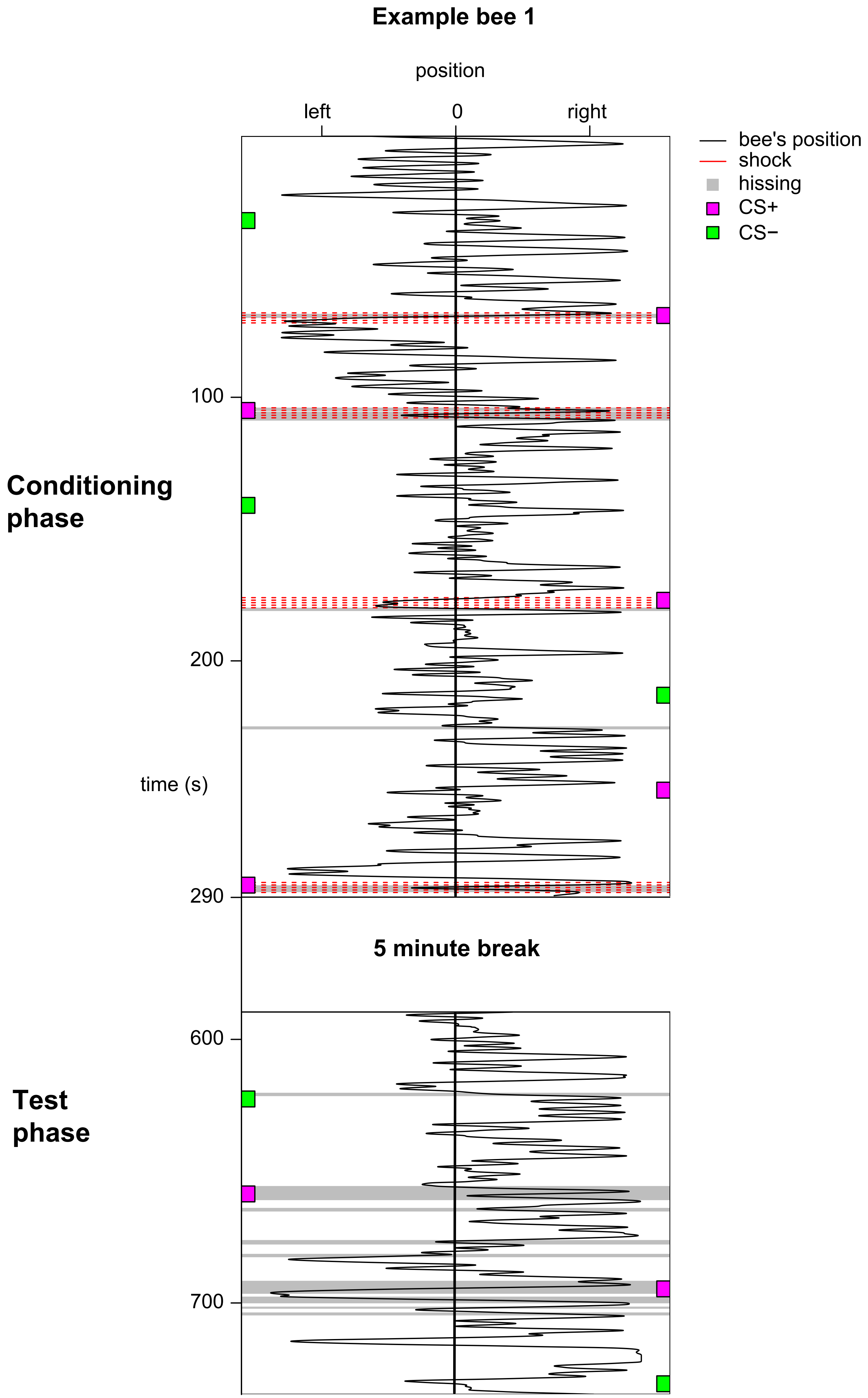
Example bee 1
position
left
0
right
bee's position
shock
hissing
CS+
CS−
100
200
290
600
700
time (s)
Conditioning phase
5 minute break
Test phase

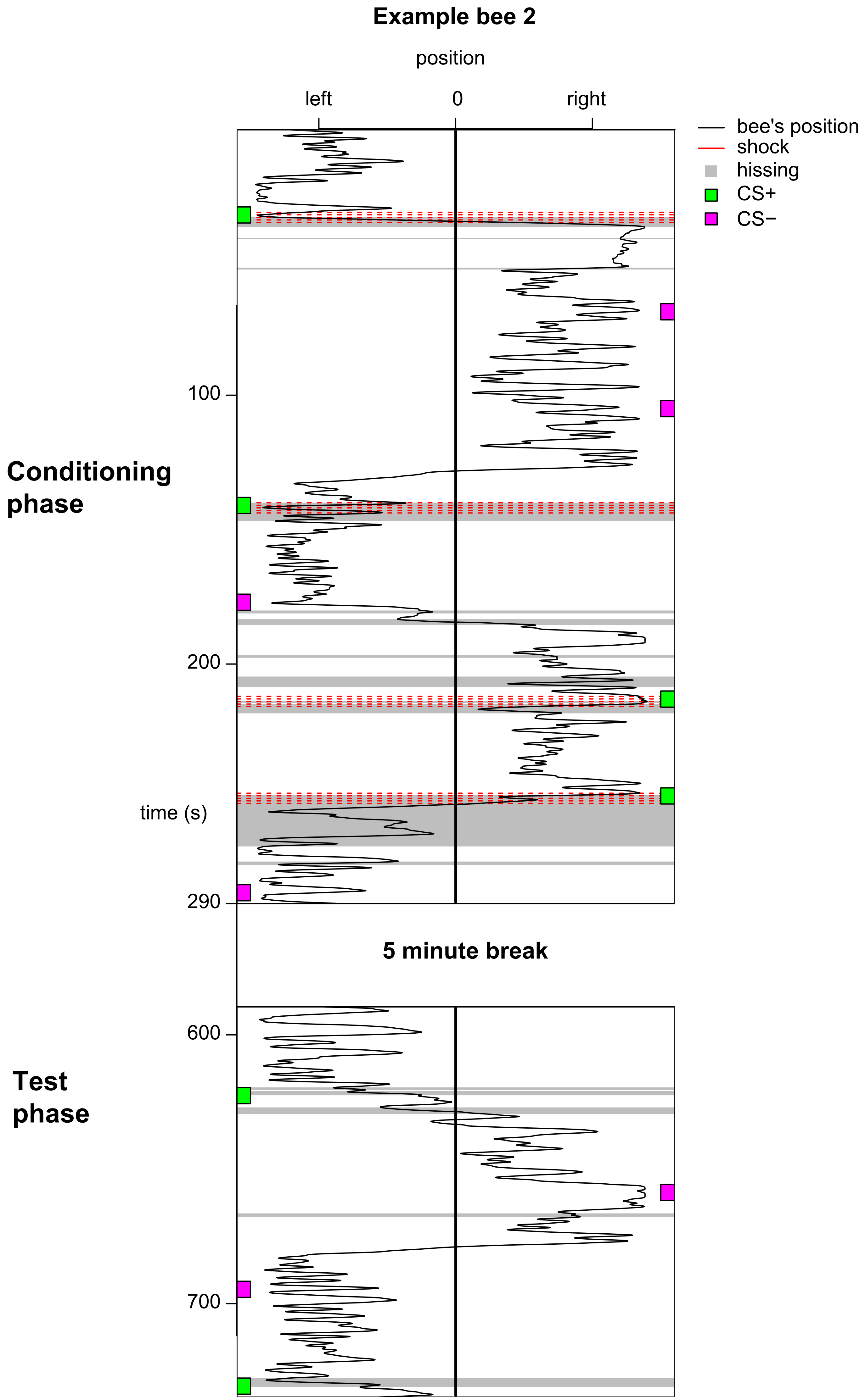

Example bee 2
position
left
0
right
bee's position
shock
hissing
CS+
CS−
100
200
290
600
700
time (s)
Conditioning phase
5 minute break
Test phase

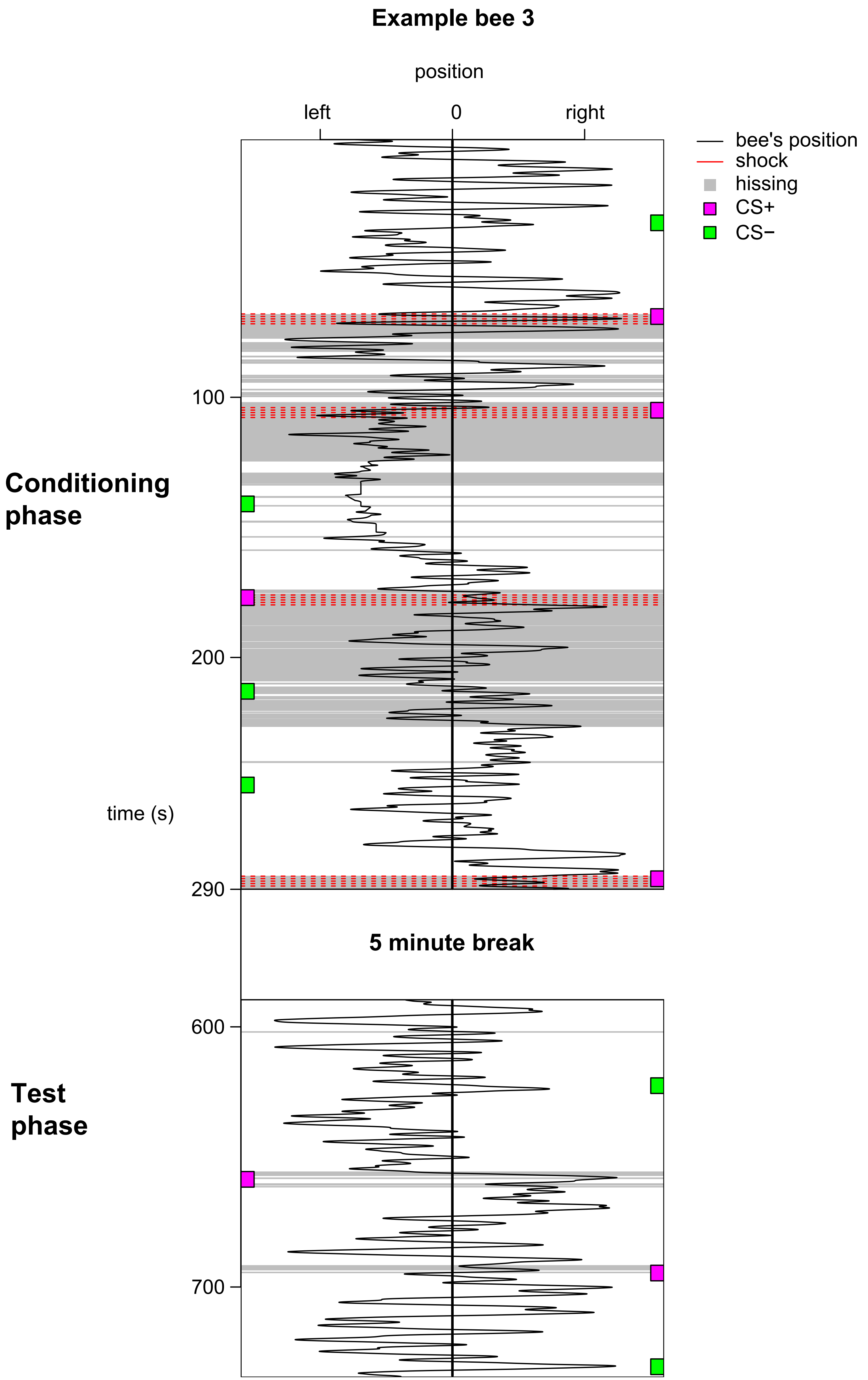
Example bee 3
position
left
0
right
bee's position
shock
hissing
CS+
CS−
100
200
290
Conditioning phase
time (s)
5 minute break
600
700
Test phase
