# Supplementary figures and images for: The Sound and the Fury—Bees Hiss when Expecting Danger
Source: PLoS One. 2015 Mar 6;10(3):e0118708. doi: 10.1371/journal.pone.0118708 (PMC4351880; doi:10.1371/journal.pone.0118708)

### Example bee 3 - spectrogram

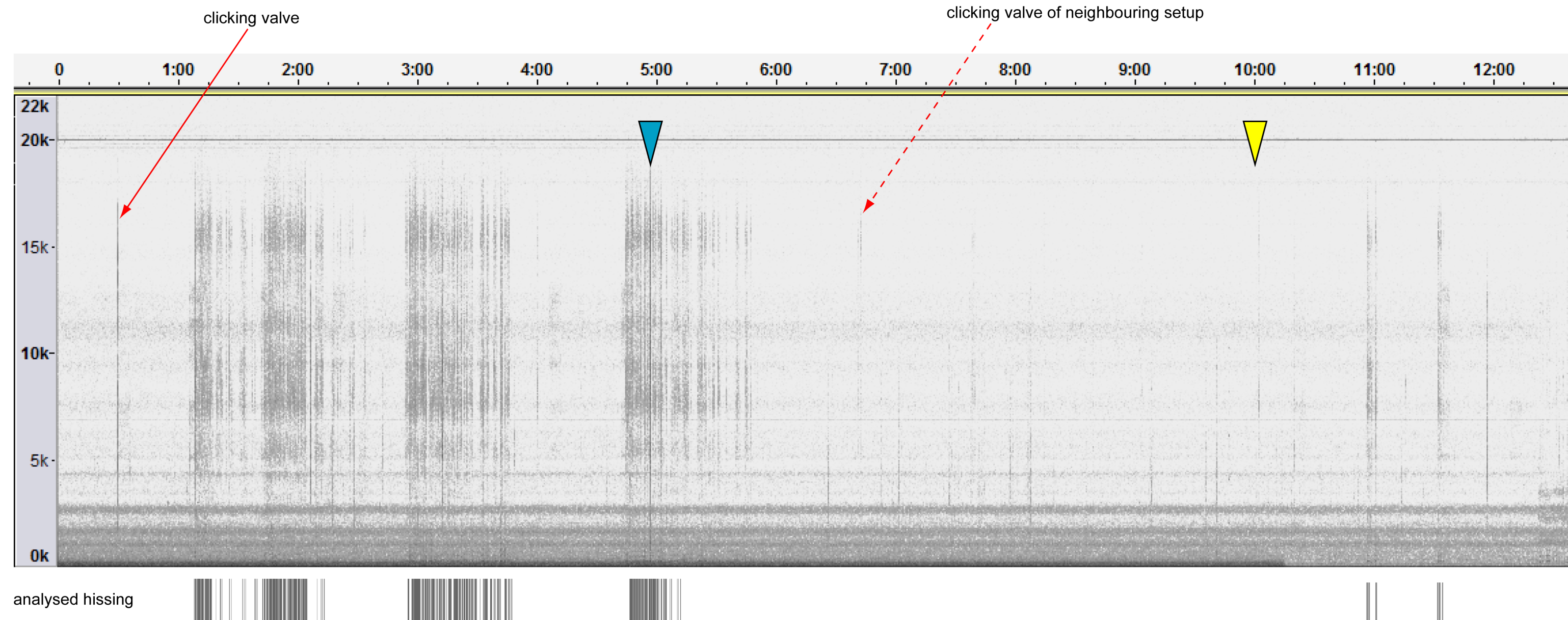

Supplement: S2 Fig — This example spectrogram belongs to the bee shown in S1c Fig. These spectrograms were used to identify hissing (see Material & Methods). The clicks of the opening and closing valves can be clearly seen as very sharp lines spanning from 0k to 18k (solid red arrow), whereas the hissing was more blurry, longer and starting only at around 4k (see trace underneath). Valves of neighbouring setups could be also recorded (dashed red arrow). Blue arrowhead marks end of conditioning, yellow arrowhead begin of recall test. (PDF) [file pone.0118708.s002.pdf]

a

**Histogram of all “hiss” lengths (bins 0.05 s)**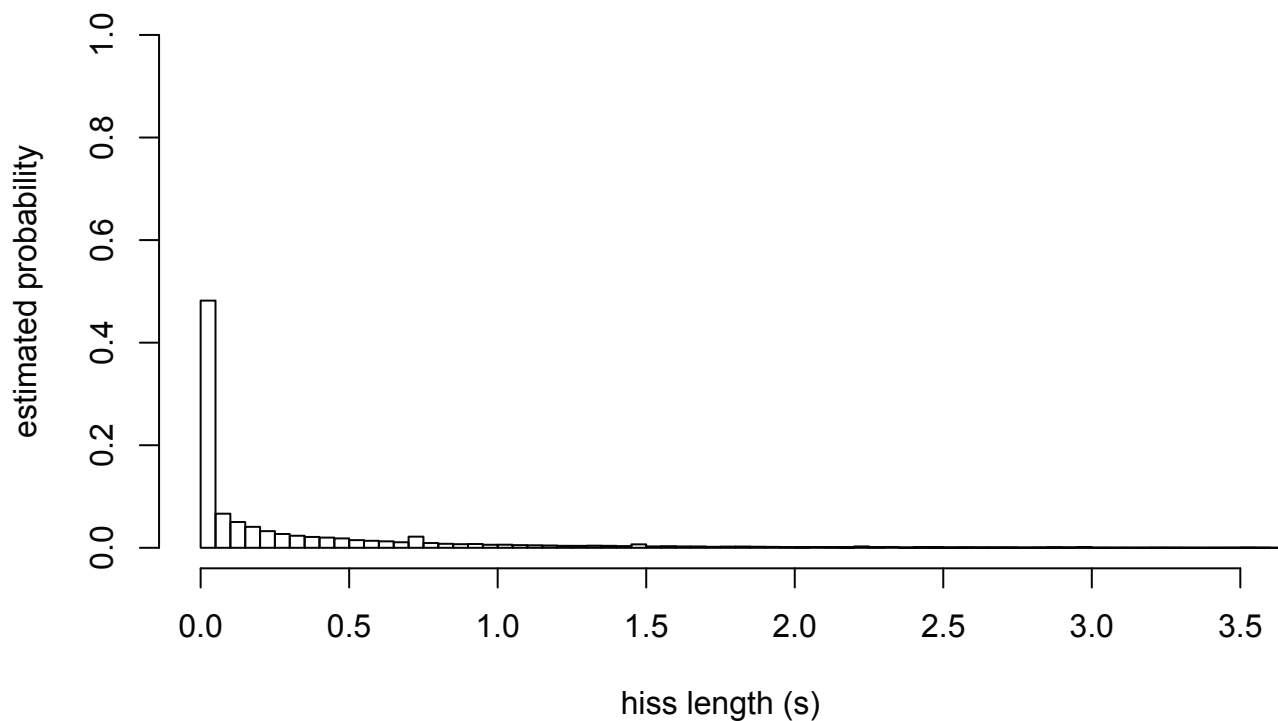

b

**Histogram of longer hiss lengths (bins 0.05 s)**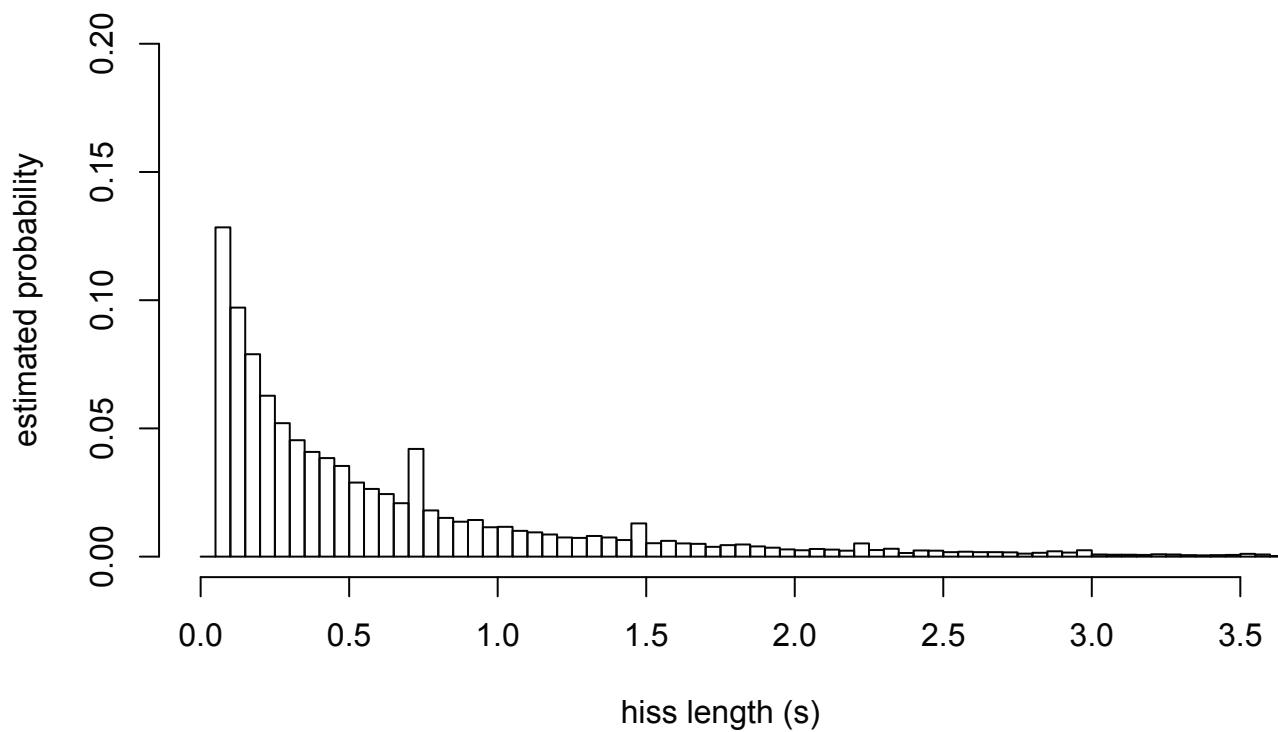

Supplement: S3 Fig — a) Histogram of the durations of all sounds recorded and initially classified as hissing before removing hisses shorter than ca. 0.09 s, which were regarded as valve clicking sounds and other events not related to bees’ hissing. b) Histogram of hissing lengths. Bin size for both plots 0.05 s. n = 104 bees. (PDF) [file pone.0118708.s003.pdf]

Hissing data of all bees

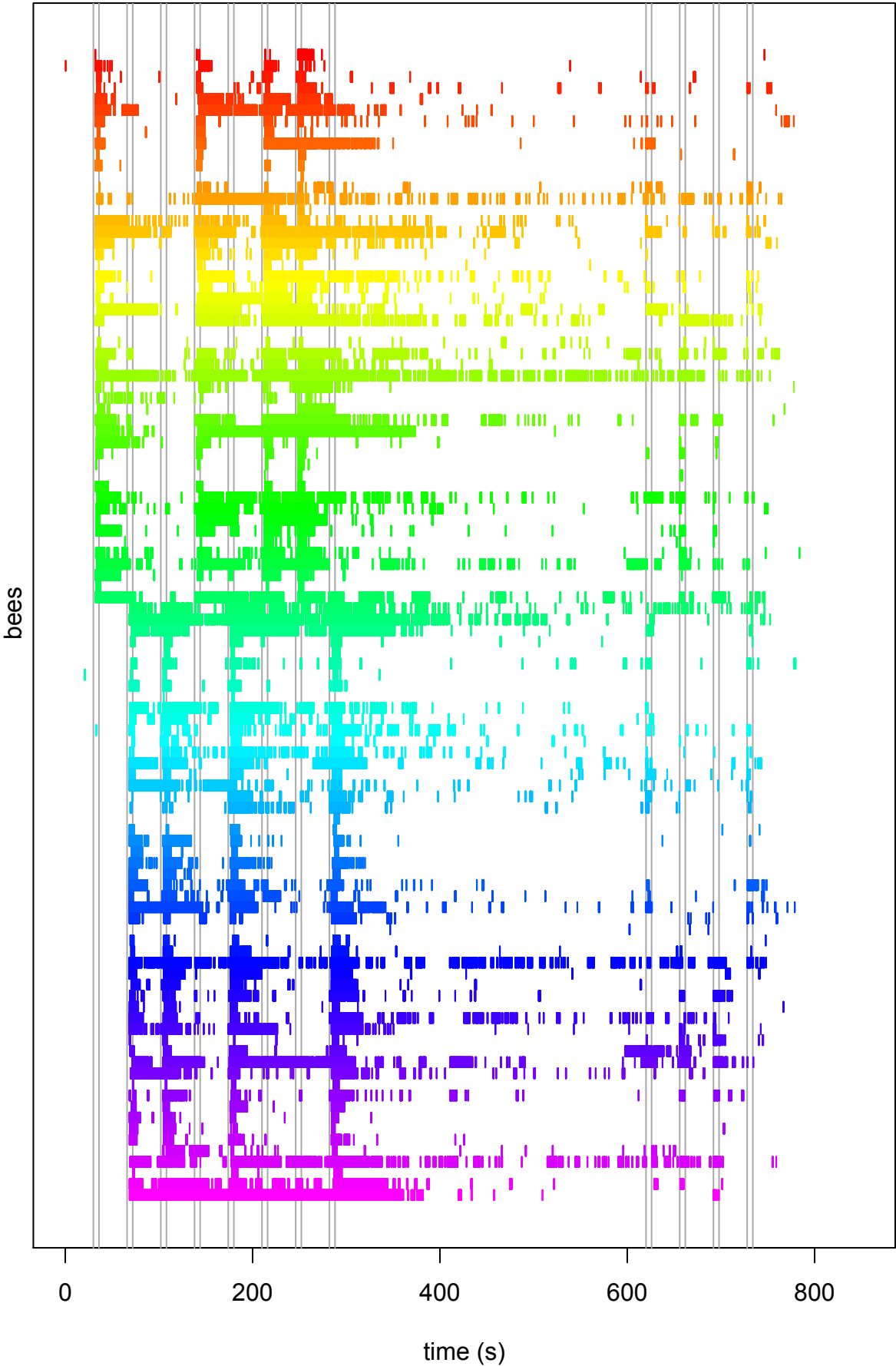

Supplement: S4 Fig — Grey double lines indicate odour presentations. In the upper part are all bees that underwent an ABBA conditioning paradigm (CS+ first), in the lower part all bees that underwent a BAAB conditioning paradigm (CS- first). Before the first CS+, few bees hiss, whereas after conditioning (300 to 600 ms), the bees’ general tendency to hiss is increased. Very few bees hissed constantly, and very few bees never hissed during the experiment (“Zombees”, one example ─ bee #26 ─ can be found at the transition from yellow to green). n = 104 bees. (PDF) [file pone.0118708.s004.pdf]

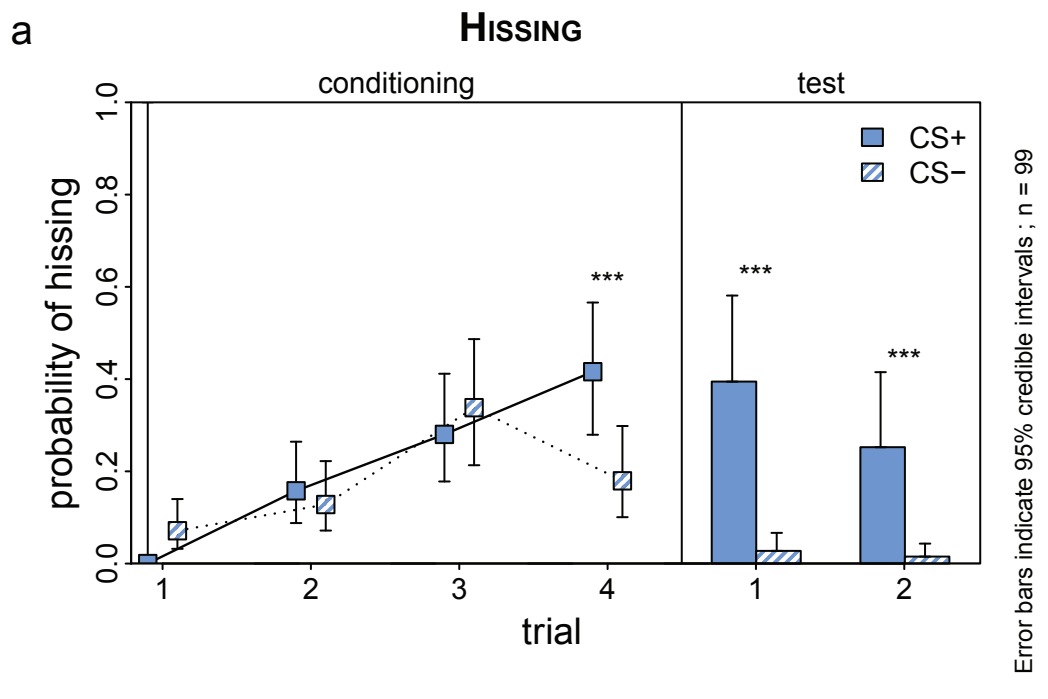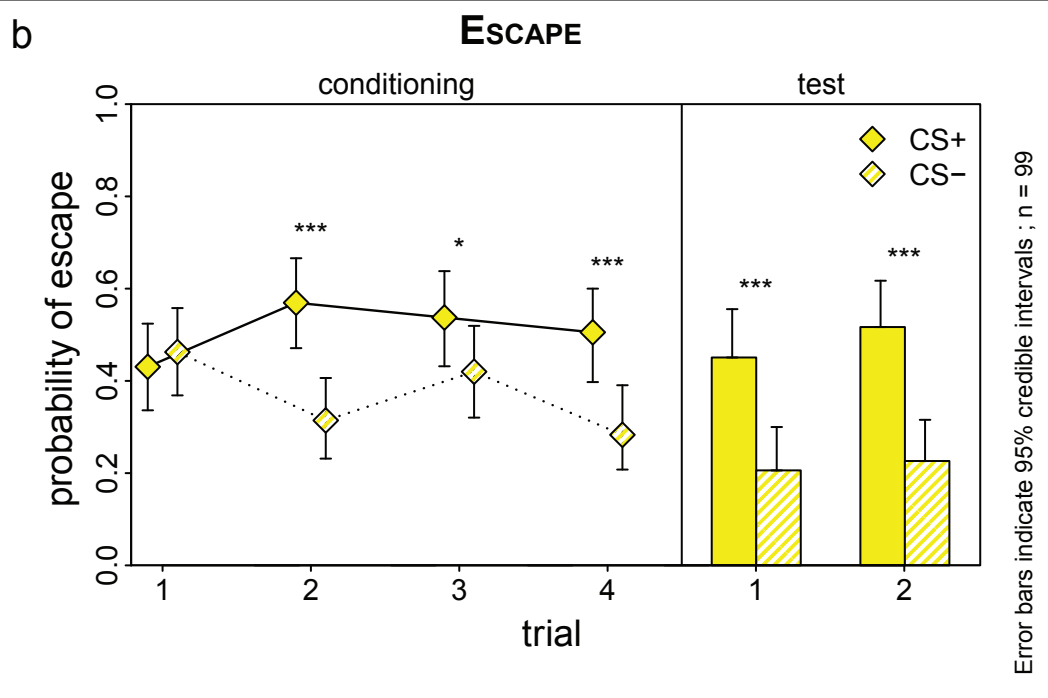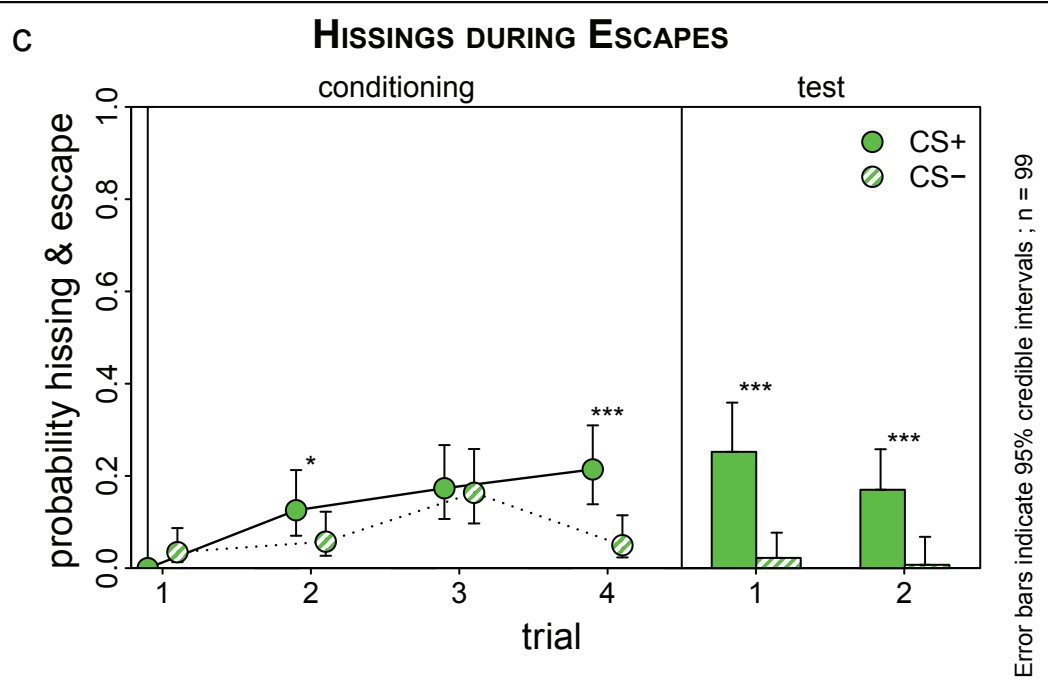

Supplement: S5 Fig — Learning studies often use ANOVA to analyse the data, however, modelling binary behavioural data is a more appropriate approach. Hissing and escape response served as binary response variables, while trial, conditioned stimulus (CS+ or CS-) and odour (hexanol or decanol) with interactions were included as fixed effects. Bee identity served as random effect to account for the repeated measurements. The credible interval for the first data point (CS+ first trial) in a) and c) spans from 0 to 1 because no observations could be made for this event. The model cannot estimate data and interval without any incident to start the estimation from. Asterisks denote statistical difference (p<0.001). (PDF) [file pone.0118708.s005.pdf]

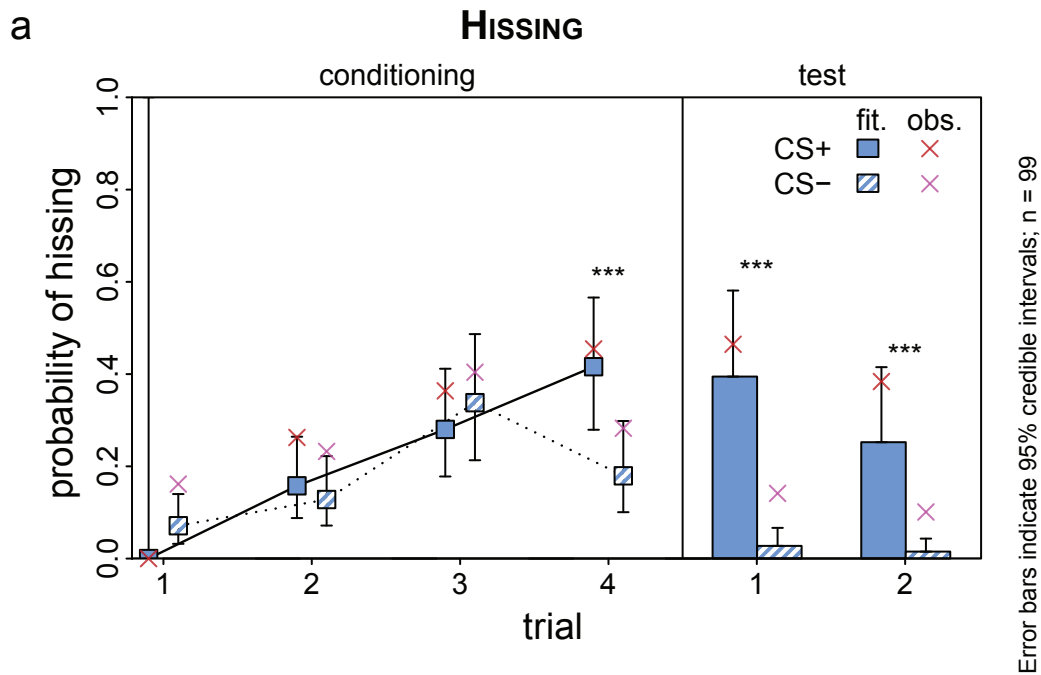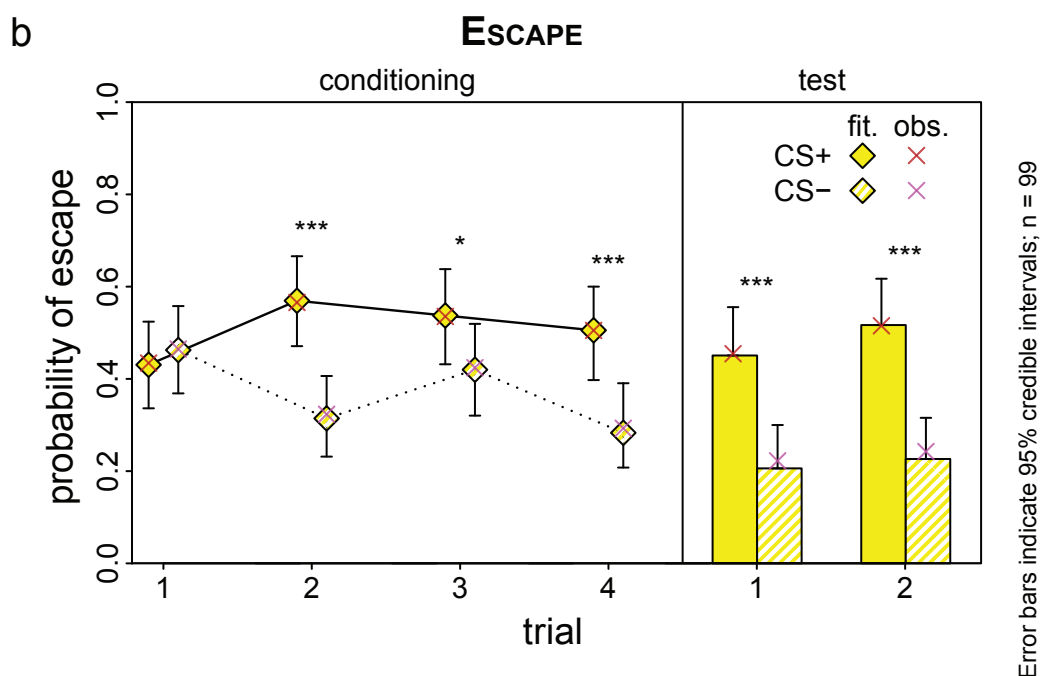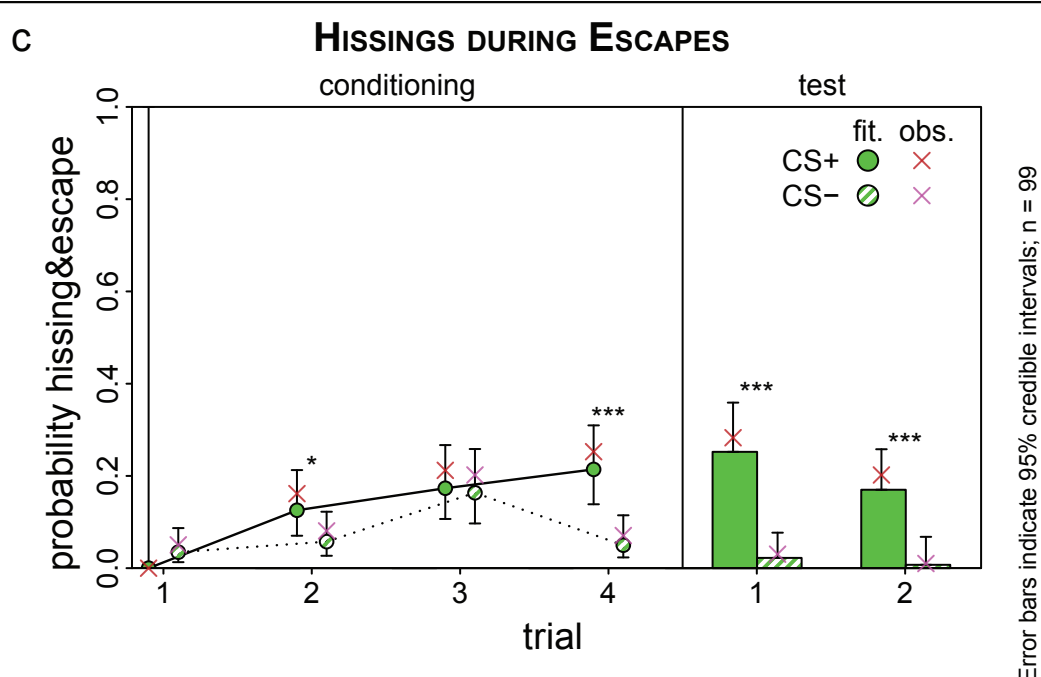

Supplement: S6 Fig — To compare the observed means (“obs., denoted by crosses) and the modelled values (“fit”), both were plotted in one figure, showing that the model generally underestimates the bees’ hissing response (see difference in a), whereas it matches the escape response (see b). Combining the two variables leads to a slight underestimation of the model compared to the observed data (c). (PDF) [file pone.0118708.s006.pdf]

a)

## Hissing

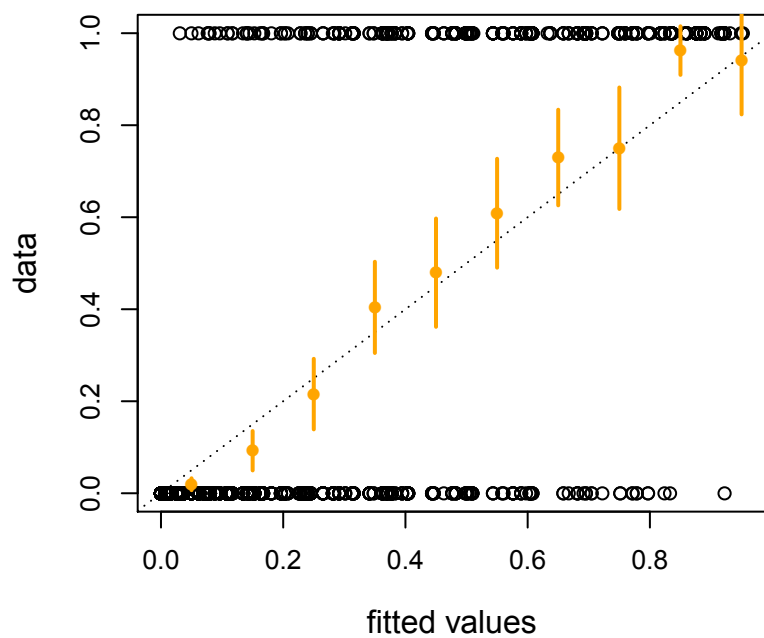

b)

## Escapes

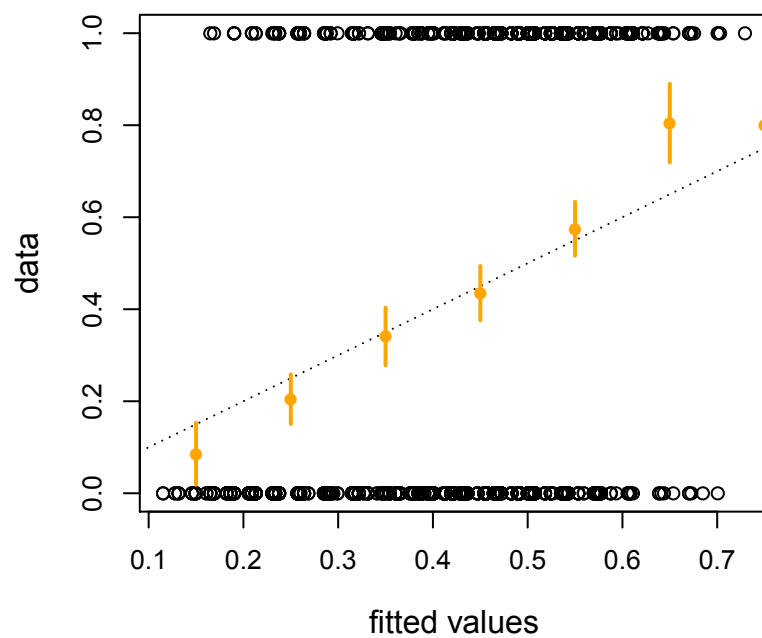

c)

## Hissing &amp; Escapes

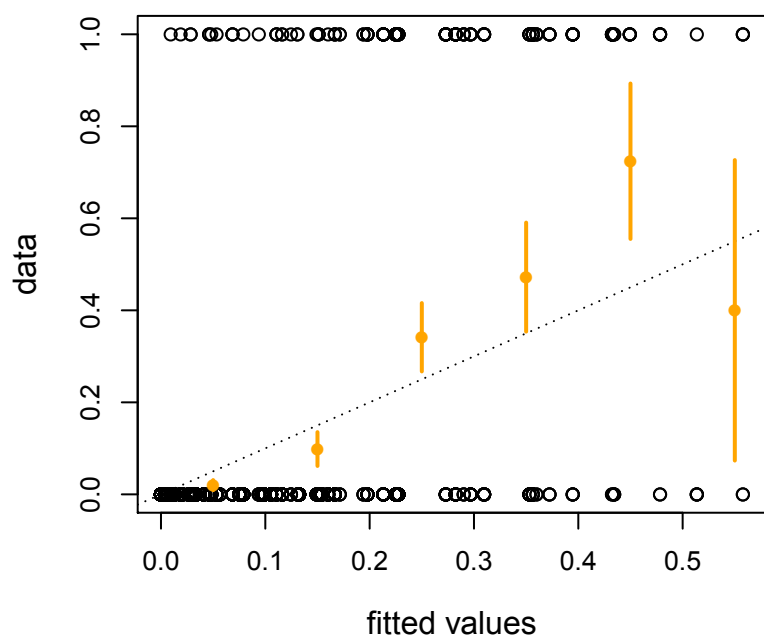

Supplement: S7 Fig — Observed vs. fitted values (open circles) are plotted with class-wise means and 95% confidence interval for the observations (orange dots and bars). The dotted line indicates perfect coincidence between the observed data and the model prediction. The confidence intervals of the class-wise means mostly span the diagonal line, which indicates that the logistic regression model predicts the data tolerably well. (PDF) [file pone.0118708.s007.pdf]

CS+: DECANOL

CS+: HEXANOL

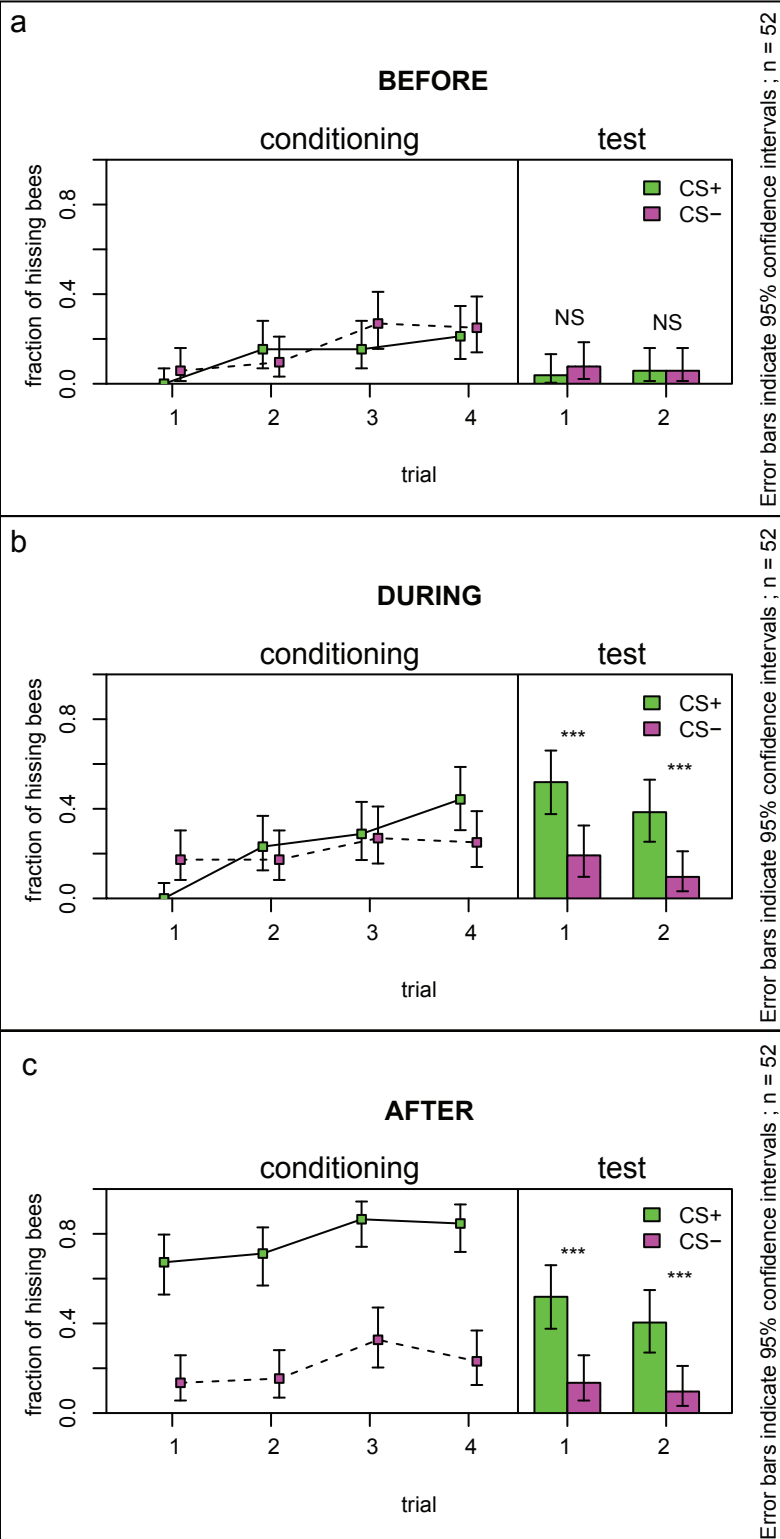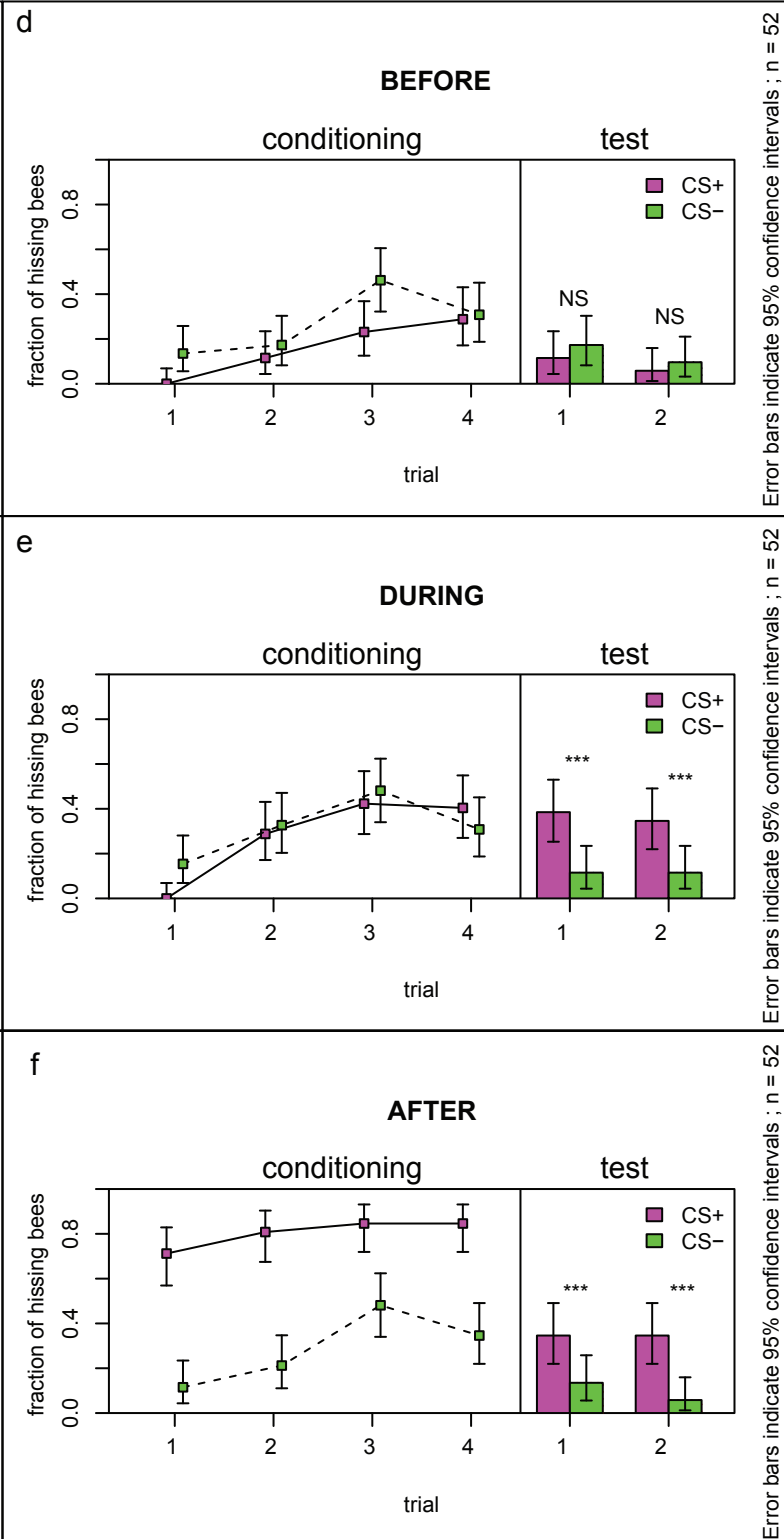

Supplement: S8 Fig — a-c) Decanol was used as CS+ and hexanol as CS-. d-f) Hexanol was used as CS+ and decanol as CS-. There was no statistically significant difference between the two odour configurations detectable. (PDF) [file pone.0118708.s008.pdf]

## honey bee responses

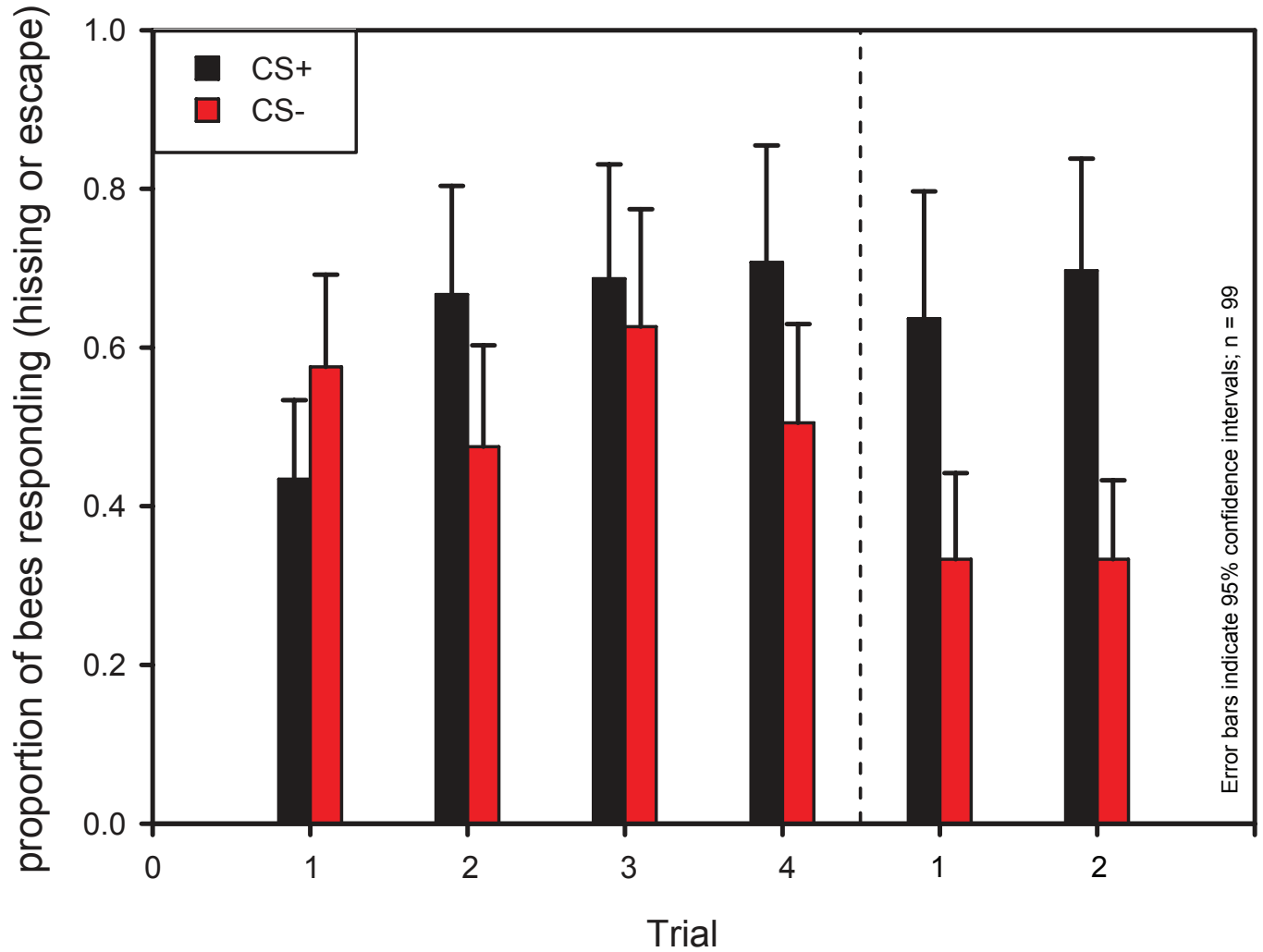

Supplement: S10 Fig — During the test, responses to the CS+ are higher as compared to the CS-. Error bars denote 95% confidence interval. (PDF) [file pone.0118708.s010.pdf]

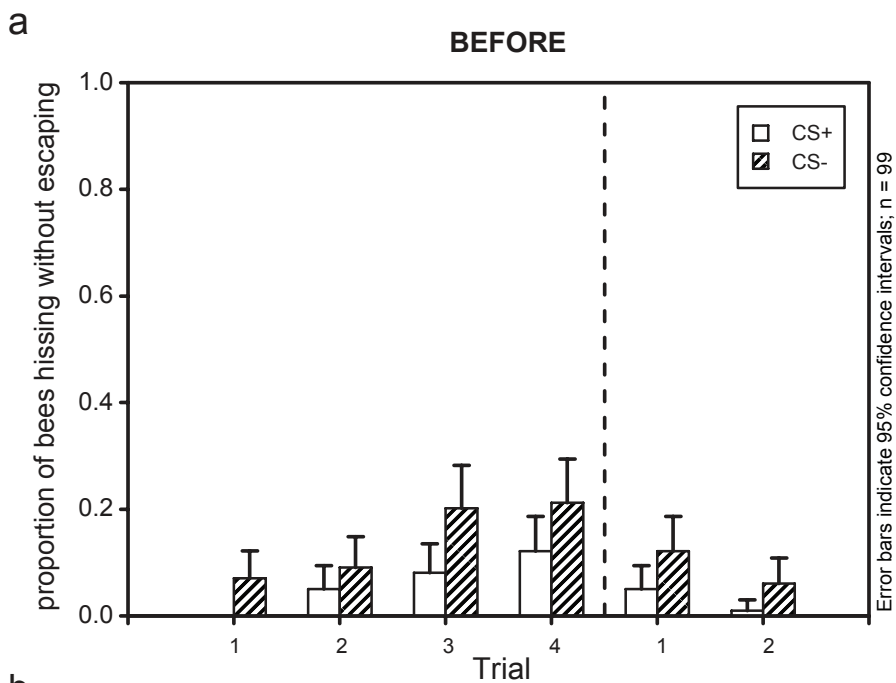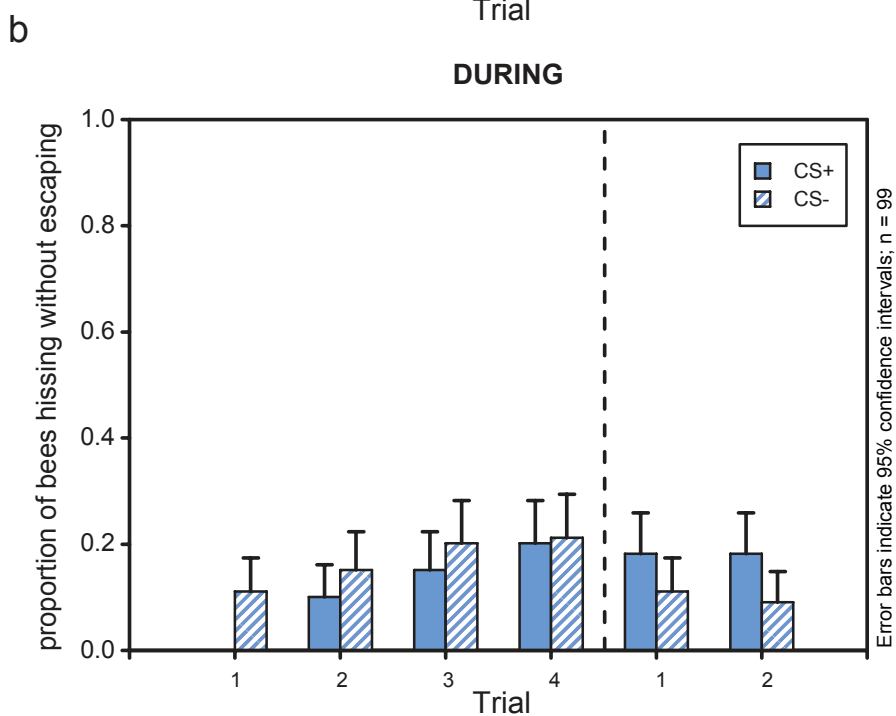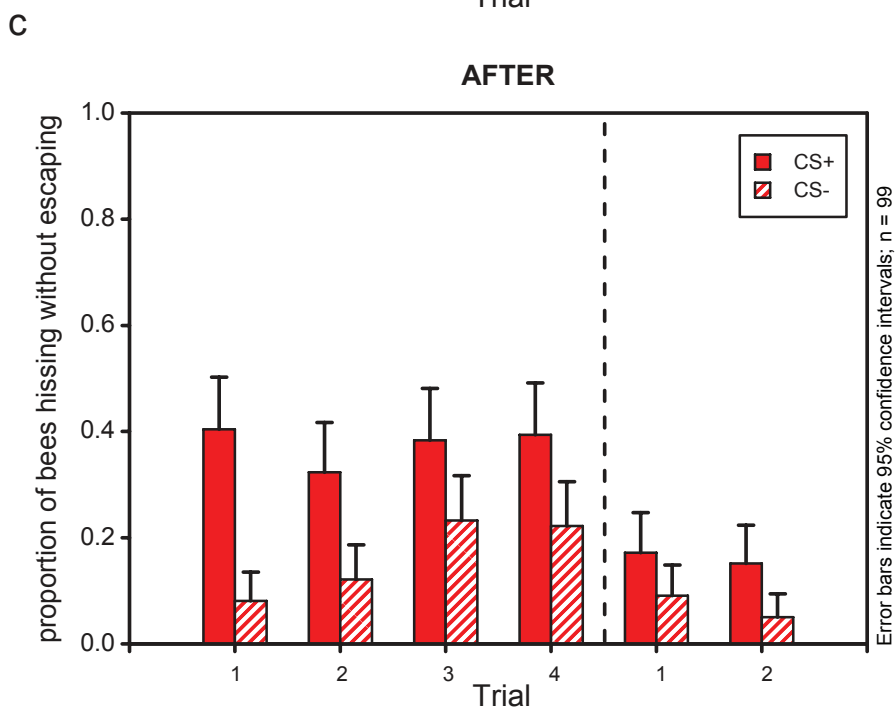

Supplement: S11 Fig — Error bars denote 95% confidence interval. (PDF) [file pone.0118708.s011.pdf]

a

## hissing time/duration plot for all CS+

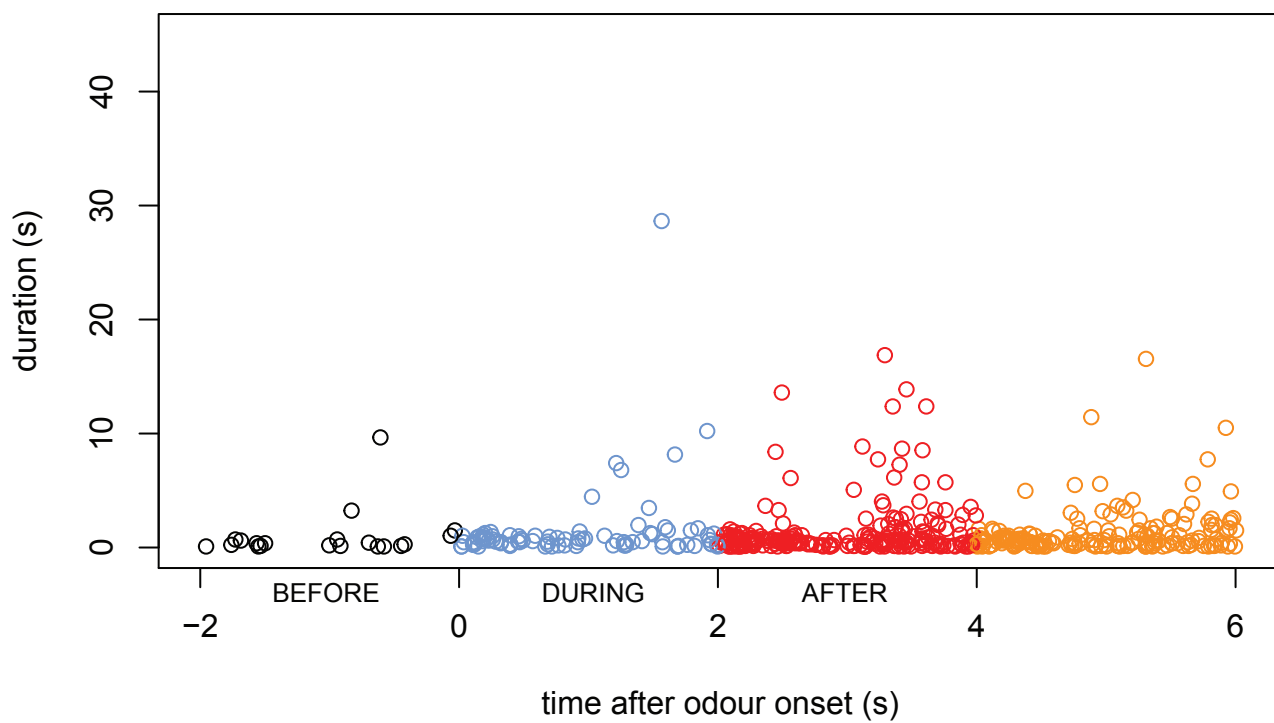

b

## hissing time/duration plot for all CS-

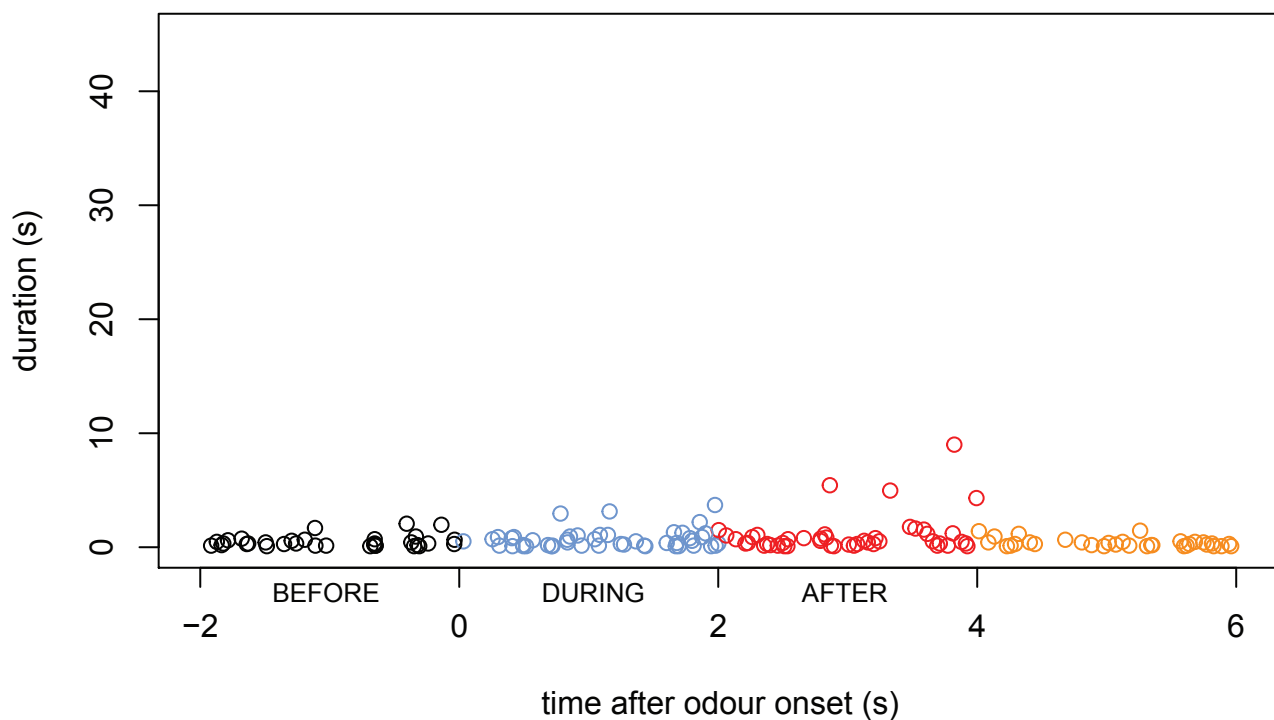

Supplement: S12 Fig — Most hisses are less than 1 s long, only very few hisses are longer than 10 seconds. Duration increases (not very surprisingly) with shock onset. n = 104 bees. (PDF) [file pone.0118708.s012.pdf]
