# Supplementary material for: The Sound and the Fury—Bees Hiss when Expecting Danger
Source: PLoS One. 2015 Mar 6;10(3):e0118708. doi: 10.1371/journal.pone.0118708 (PMC4351880; doi:10.1371/journal.pone.0118708)
Supplement: S9 Fig — No statistically significant differences could be found when analysing the bees’ escape behaviour with respect to the odour configuration. Bees which were conditioned to decanol as CS+ (a) as well as bees that were conditioned to hexanol as CS+ (b) distinguished between CS+ and CS- after conditioning to the same extent. (PDF) [file pone.0118708.s009.pdf]

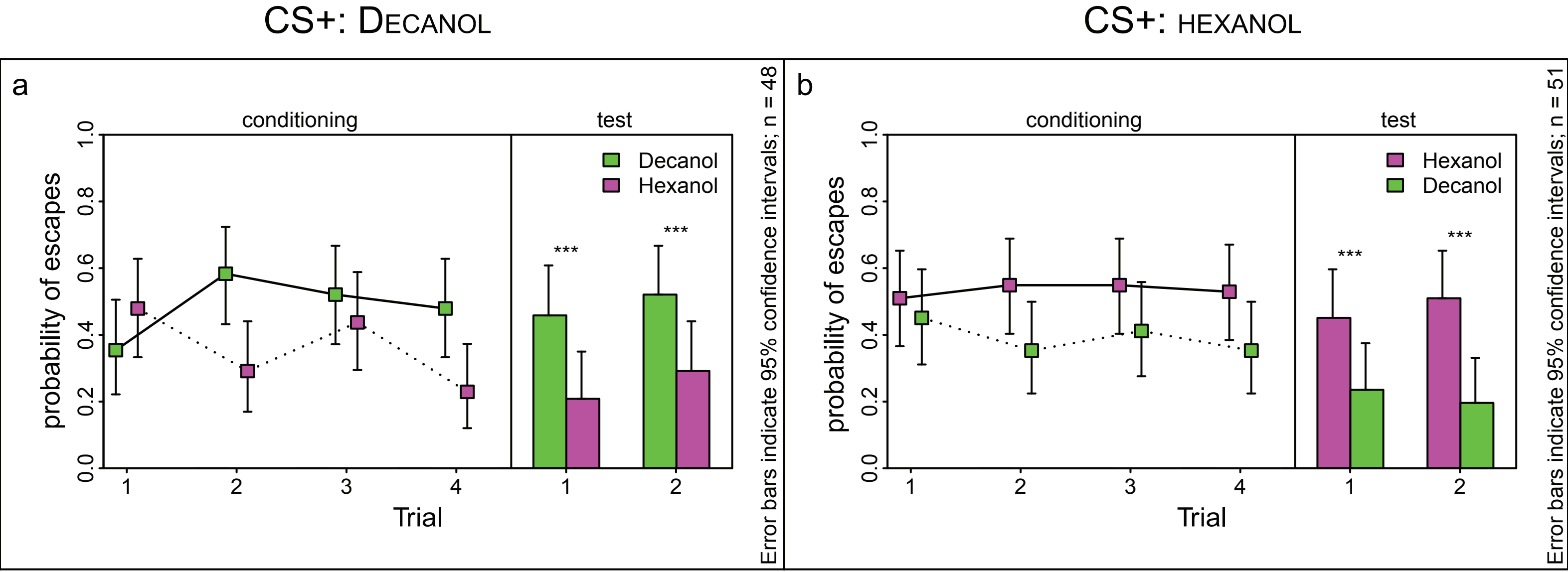

CS+: DECANOL
CS+: HEXANOL
a
conditioning
test
probability of escapes
0.0
0.2
0.4
0.6
0.8
1.0
Decanol
Hexanol
***
***
1
2
3
4
1
2
Trial
Error bars indicate 95% confidence intervals; n = 48
b
conditioning
test
probability of escapes
Hexanol
Decanol
***
***
Trial
Error bars indicate 95% confidence intervals; n = 51
